# Organic Synaptic Transistors and Printed Circuit Board Defect Inspection with Photonic Stimulation: A Novel Approach Using Oblique Angle Deposition

**DOI:** 10.1002/smll.202501997

**Published:** 2025-05-07

**Authors:** Gyeongho Lee, Yeo Eun Kim, Hyeonjung Kim, Han‐Koo Lee, Jae Yeon Park, Seyong Oh, Hocheon Yoo

**Affiliations:** ^1^ Semiconductor Total Solution Center Korea Institute of Ceramic Engineering and Technology 3321 Gyeongchung‐daero Icheon 17303 Republic of Korea; ^2^ Department of Materials Science and Engineering Korea University 145 Anam‐ro Seoul 02841 Republic of Korea; ^3^ Department of Semiconductor Engineering Gachon University 1342 Seongnam‐daero Seongnam 13120 Republic of Korea; ^4^ Division of Electrical Engineering Hanyang University ERICA 55 Hanyangdaehak‐ro Ansan 15588 Republic of Korea; ^5^ Pohang Accelerator Laboratory Pohang University of Science and Technology (POSTECH) 80 Jigok‐ro Pohang 37673 Republic of Korea; ^6^ Radiation Fusion Technology Research Division Advanced Radiation Technology Institute (ARTI)/Korea Atomic Energy Research (KAERI) 29 Geum gu‐gil Jeongeup 56212 Republic of Korea; ^7^ Department of Electronic Engineering Hanyang University 222 Wangsimni‐ro Seoul 04763 Republic of Korea

**Keywords:** defect inspection, morphology engineering, oblique angle deposition, optoelectronic synapse, parallel potentiation‐depression

## Abstract

This study introduces a photonic stimulation‐based synaptic transistor utilizing oblique angle deposition (OAD) of dinaphtho[2,3‐b:2′,3′‐f]thieno[3,2‐b]thiophene (DNTT). While OAD enables advanced nanostructures, its application to organic materials remains largely unexplored. Here, the electrical characteristics and photoinduced trap behavior of obliquely deposited DNTT transistors are systematically investigated, successfully replicating key synaptic functions. OAD‐controlled grain size and spacing in the DNTT channel yield distinct performance metrics compared to conventional devices. The introduced trap regions enable stable synaptic behavior across diverse gate voltage (*V_G_*) conditions. By adjusting presynaptic photonic pulse intensity, duration, and repetition, a robust transition is achieved to long‐term memory (LTM). The device further demonstrates reliable optoelectronic synaptic operation over 52 durability cycles. Concurrent photonic stimulation enables parallel potentiation‐depression dynamics, enhancing processing speed and performance, highlighting its promise for next‐generation neuromorphic computing. Its application is also showed in printed circuit board (PCB) defect inspection, successfully mimicking biological synapses under simultaneous photonic stimulation.

## Graphical abstract

Photonic stimulation‐based synaptic transistor using obliquely deposited dinaphtho[2,3‐b:2',3'‐f]thieno[3,2‐b]thiophene (DNTT) is proposed. The device exhibits stable, tunable optoelectronic synaptic behaviors due to controlled nanostructures and trap dynamics, enabling long‐term memory (LTM) and parallel processing. Its potential is validated by means of PCB defect inspection mimicking biological synapses.

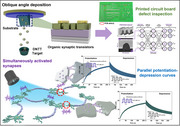

## 1 Introduction

Oblique angle deposition (OAD), also known as glancing angle deposition (GLAD), is a powerful technique for fabricating nanostructured films by directing material flux at an oblique angle onto a substrate in a vapor deposition system.^[^ ^1^, ^2^ ^]^ This approach enables morphology engineering by forming diverse nanostructures, including columns, helices, and zigzags, through shadowing effects controlled by deposition parameters.^[^ ^3^, ^4^, ^5^, ^6^ ^]^ Particularly, these nanostructures formed through OAD enhance interactions with charged or polarized species, such as electrons, holes, ions, and molecules, increasing the number of reactive sites.^[^ ^7^ ^]^ While OAD has been extensively explored with inorganic semiconductors and metals, which have demonstrated significant applications in sensors,^[^ ^8^, ^9^, ^10^ ^]^ solar cells,^[^ ^11^, ^12^ ^]^ antireflective coatings,^[^ ^13^, ^14^ ^]^ and microsupercapacitors,^[^ ^14^, ^15^ ^]^ its potential with organic materials remains underexplored.

Organic semiconductors (OSCs) are being actively explored through development of new materials and modification of properties for applications such as organic field‐effect transistors (OFETs),^[^ ^16^, ^17^, ^18^, ^19^, ^20^ ^]^ organic light‐emitting diodes^[^ ^21^, ^22^ ^]^ (OLEDs), organic photovoltaic (OPV) devices,^[^ ^23^, ^24^ ^]^ organic photodetectors (OPDs),^[^ ^25^, ^26^ ^]^ and organic electrochemical transistors (OECTs).^[^ ^27^, ^28^ ^]^ Among these approaches, OAD‐based morphology engineering, characterized by its low‐temperature processing and vacuum deposition system, is highly suitable for OSCs due to its ability to strategically modulate electrical or optical properties.^[^ ^29^, ^30^, ^31^ ^]^ For example, Kim et al. reported enhanced OFET mobility due to the superior morphological properties of the OSC formed along the temperature gradient during OAD.^[^ ^32^ ^]^ Furthermore, OSCs typically have a narrower bandgap than inorganic semiconductors, allowing efficient interaction with a broader light spectrum.^[^ ^33^, ^34^ ^]^ This suggests that OAD can enable unique emergent functions, such as photoinduced charge trapping.

In parallel, optoelectronic synaptic devices have gained attention as a foundation for neuromorphic computing, offering an alternative to the von Neumann architecture.^[^ ^35^, ^36^, ^37^, ^38^, ^39^ ^]^ A key advantage of optoelectronic devices is their ability to leverage the uniformity of light for simultaneous stimulation of multiple synaptic units, enabling parallel synaptic operations.^[^ ^40^, ^41^ ^]^ For example, Luan et al. reported manifold synaptic characteristics and controllability of synaptic weights, including excitatory postsynaptic current (EPSC), paired pulse facilitation (PPF), and spike‐number dependent plasticity (SNDP), through a near‐infrared (NIR) photoresponse of Yb/Ho upconversion quantum dots (UCQDs).^[^ ^42^ ^]^ This contrasts with traditional electronic synaptic devices, which often suffer from signal degradation and high‐power consumption due to sequential electrical input requirements.^[^ ^43^, ^44^, ^45^ ^]^ By integrating optics and electronics into a single device, optoelectronic synaptic systems achieve superior controllability of synaptic weights and expansive bandwidth with faster data processing speeds.^[^ ^36^, ^46^, ^47^, ^48^ ^]^ The incorporation of OAD techniques with organic semiconductors further enhances these capabilities by enabling the creation of tailored nanostructures optimized for light‐matter interactions. This synergy positions OAD‐based organic optoelectronic synaptic devices as a transformative approach for efficient, large‐scale neuromorphic computing.

**Figure 1:**
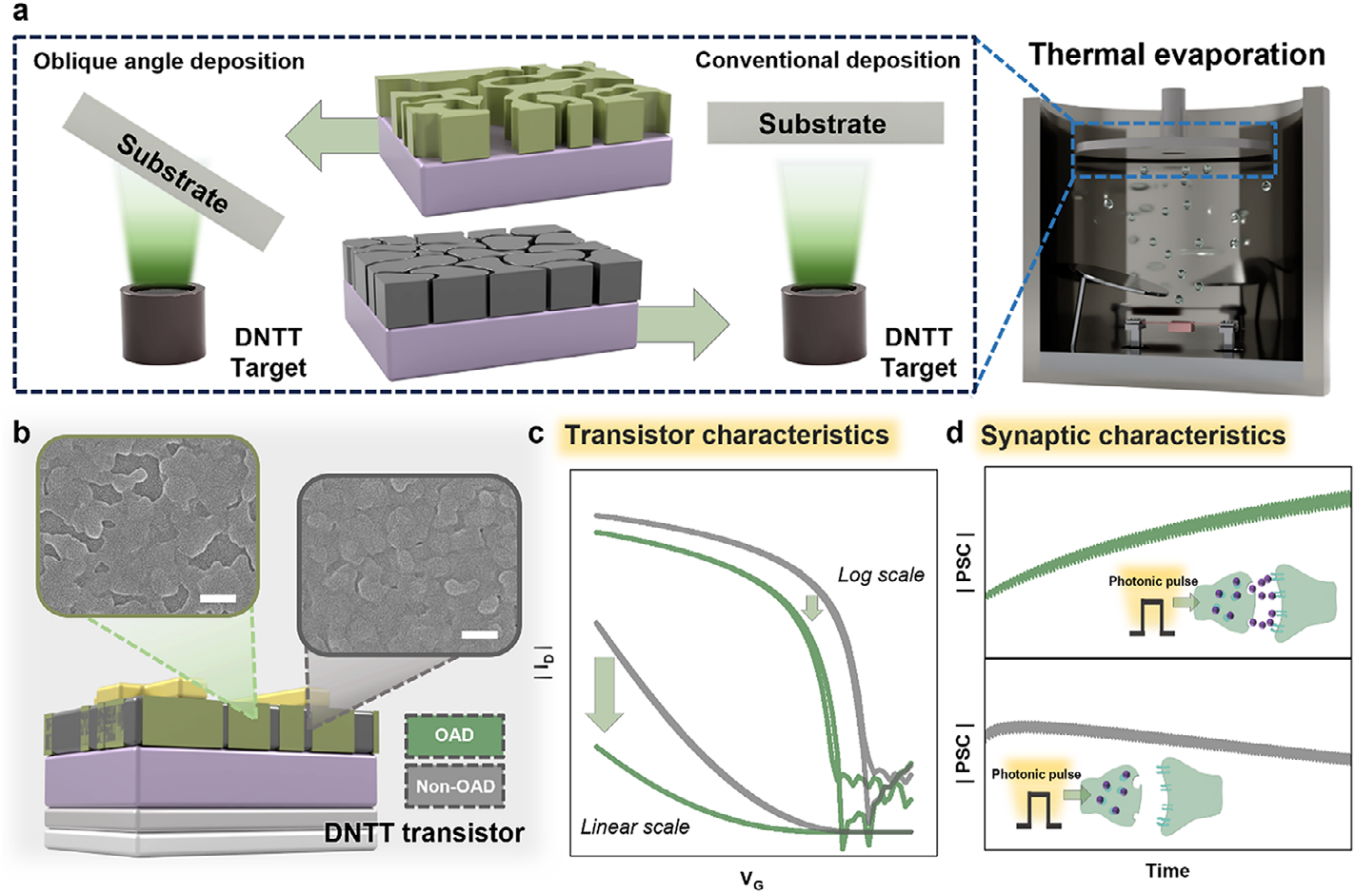
a) Schematic illustration of the OAD and conventional deposition processes via thermal evaporation and the resulting morphologies of the DNTT thin film. b) Top view SEM images showing difference in DNTT grains depending on OAD (scale bar = 200 nm). c) Changes in transistor characteristics and d) differences in synaptic characteristics with and without OAD.

In this study, we present a strategy for engineering the morphology of OSCs using a physical vapor deposition (PVD) system (**Figure** **1**a). The DNTT layer fabricated via OAD exhibits warped grains with pronounced gaps, in contrast to the uniform grain morphology produced by conventional deposition methods. Based on this distinct morphological difference, we fabricated DNTT transistors with and without OAD application (Figure 1b). The resulting transistor characteristics were systematically analyzed, revealing significant performance variations (Figure 1c). Crucially, rather than conventional synaptic transistors that often rely on added materials or complex structures such as heterostructures, blends, or floating gates to enable charge trapping,^[^ ^49^, ^50^, ^51^ ^]^ The OAD‐modified DNTT channel successfully induced EPSC by photonic stimulation, providing structural simplicity to the device (Figure 1d). Additionally, the gate voltage (*V_G_*) tunability of synaptic characteristics was demonstrated, enabling control over synaptic weights and the linearity of long‐term potentiation (LTP) across various *V_G_* values. This comprehensive analysis underscores the impact of OAD‐engineered DNTT morphology on transistor performance and synaptic behavior. Furthermore, global photonic stimulation (i.e., simultaneously applying to multiple devices) facilitated parallel potentiation‐depression dynamics, allowing printed circuit board (PCB) defect inspection. These findings highlight the potential of OAD‐based morphology control in advancing OSC devices for neuromorphic and optoelectronic applications.

## 2 Results and Discussion

**Figure 2:**
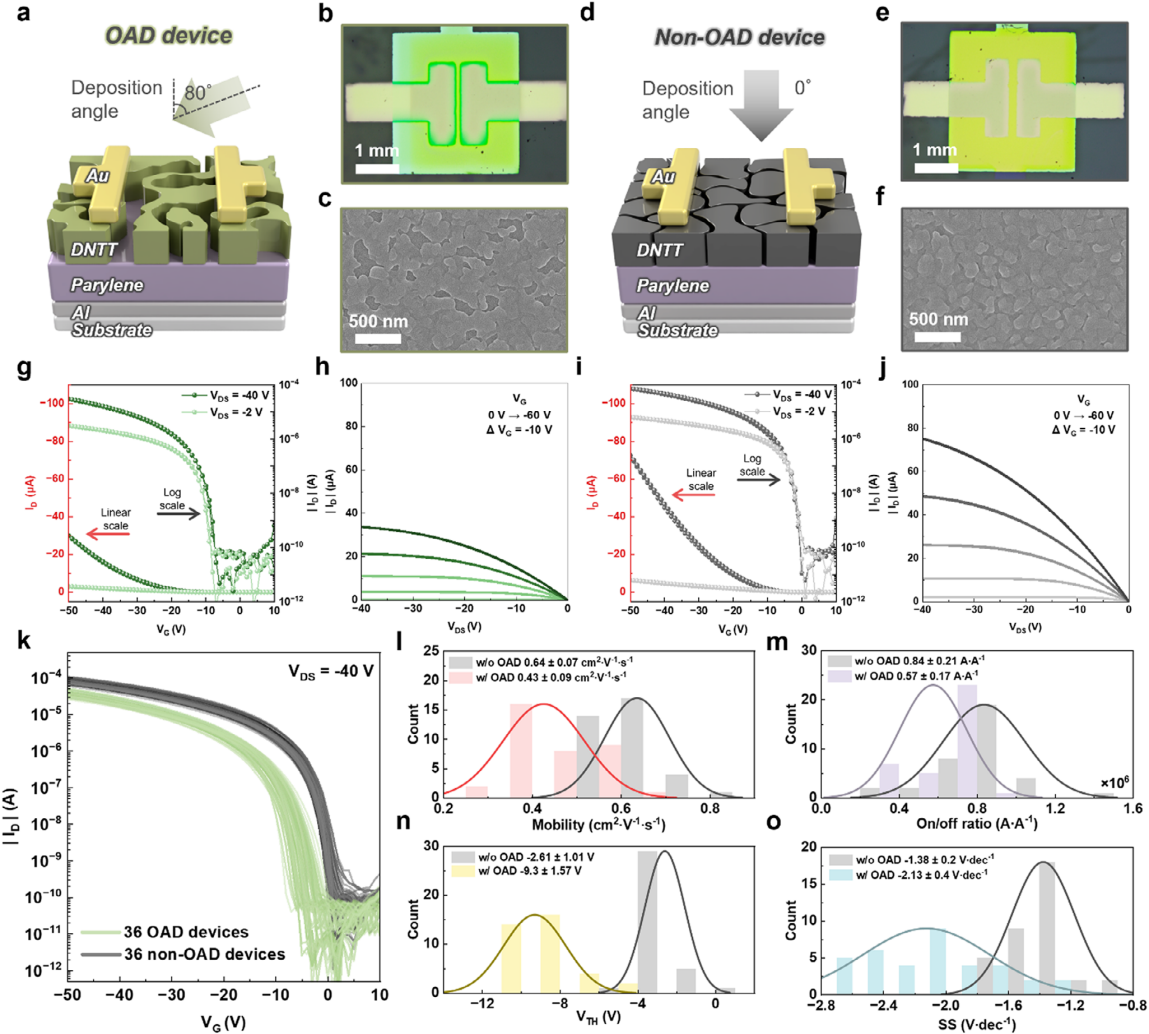
For the OAD device, a) deposition angle and configuration, b) OM image and c) top view SEM image. For the non‐OAD device, d) deposition angle and configuration, e) OM image and top view SEM image. g) Transfer curve and h) output curve of the OAD device. i) Transfer curve and j) output curve of the non‐OAD device. k) 36 transfer curves for each device with and without OAD. Extracted electrical parameters from 36 transfer curves of each device with and without OAD: l) mobility, m) on/off ratio, n) *V_TH_*, and o) *SS* statistics, including mean values and standard deviations.

We fabricated two types of morphological‐dependent transistors (i.e. OAD and non‐OAD devices), distinguished by the presence or absence of OAD on the DNTT channel. **Figure** **2**a depicts the configuration of the OAD device with a DNTT layer obliquely deposited at 80°. Aluminum (Al) and Parylene were used as the gate and gate dielectric, respectively. The top view scanning electron microscopy (SEM) image of the OAD device reveals that the DNTT grains were large and exhibited warped spacings between them (Figure 2c). In contrast, as illustrated in Figure 2d, the structure of the non‐OAD device with a deposition angle of 0° exhibited relatively smaller grain sizes and a more compact grain arrangement, as evidenced by the top view SEM image in Figure 2f. Here, the DNTT morphology was significantly altered by OAD to control the formation of interfacial trap regions at the junction between the DNTT channel and Parylene. This morphological difference between the devices was also visually apparent in the optical microscopy (OM) images (Figure 2b,e).

Next, the transistor characteristics of OAD and non‐OAD devices were investigated to confirm the effects of morphological modifications of DNTT, as shown in Figure 2g–j. In the transfer curve, the OAD device turned on at −6 V with a drain‐source voltage (*V_DS_*) of −40 V, and the drain current (*I_D_*) was 33.8 µA at a given condition (*V_DS_* = −40 V and *V_G_* = −60 V) in the output curve. In comparison, the non‐OAD device began to turn on at 0 V, with an *I*_*D*_ of 75.2 µA at *V_DS_* = −40 V and *V_G_* = −60 V. Moreover, lower I_D_ and turn‐on voltage (*V_ON_*) shifts were also shown within the linear region of the transfer curve (*V_DS_* = −2 V) (Figure 2g,i). OAD induced variations in fundamental transistor characteristics such as *V_ON_* and *I_D_* by morphological alterations in the DNTT channel.

To ascertain the reliable effects of OAD on transistor characteristics, 36 transistors were fabricated for each device, with and without OAD applied and the transfer curves were measured (Figures S1 and S2, Supporting Information). Figure 2k presents transfer curve profiles that display distinctive trends consistent with the aforementioned electrical characteristics in Figure 2g,i. Thereafter, to characterize the variation between OAD and non‐OAD devices, values of mobility (*μ*), on/off ratio, threshold voltage (*V_TH_*), and subthreshold swing (*SS*) were extracted for each of the 36 transistors. The charge *μ* and *SS* were calculated using the following equations:

(1) $$\mu \;\left(cm^{2}\cdot V^{-1}\cdot s^{-1}\right)=\frac{dI_{D}}{dV_{G}}\;\cdot \frac{L}{WC_{ox}V_{DS}}$$

where *L* and *W* represent the channel length and width, respectively, while *C_ox_* denotes the dielectric capacitance of a Parylene film.

(2) $$SS\;\left(V\cdot dec^{-1}\right)=\frac{dV_{G}}{d\left(log\;I_{D}\right)\;}\;$$

*SS*, which represents the current change relative to the voltage change when the transistor becomes active, was calculated as the maximum slope of the transfer curve.

The obtained average *μ* of the OAD and non‐OAD devices was found to be 0.43 and 0.64 cm^2^ V^−1^ s^−1^, respectively (Figure 2l). The average on/off ratio of the OAD device was ≈32.8% lower than that of the non‐OAD device (Figure 2m). These results were attributed to the delayed charge transport within the channel, caused by the relatively large and warped DNTT grains formed through OAD. *V_TH_* of the OAD device shifted by an average of as much as Δ*V* = −6.69 V (Figure 2n), and switching performance was relatively poor, resulting in an increase in *SS* by 0.75 V dec^−1^ (Figure 2o). Thus, the dimensions and configuration of DNTT grains modified by OAD significantly influenced the electrical characteristics of the transistor by reducing hole concentration in the channel, leading to decreased performance. On the other hand, there are also compelling advantages: particularly in enhancing synaptic characteristics through the photogating effect. Photogating occurs when carriers generated by light with wavelengths exceeding the material's bandgap become trapped, resulting in a shift in *V_TH_*.^[^ ^52^, ^53^ ^]^ This effect induces hysteresis in the transfer characteristics, evident between the forward and reverse sweeps. By analyzing the photoresponse of the OAD‐modified DNTT device, we confirmed the presence of photogating through the formation of trap regions.

To confirm the consistent reliability of fabrication process for the OAD and non‐OAD devices, the batch‐to‐batch variability was investigated. Figure S3 (Supporting Information) shows optical microscope (OM) images with the measured 36 transistors of the OAD and non‐OAD devices, respectively. A total of 36 transistors were characterized by measuring 18 devices in the red or blue regions from each die of the OM images. The uniform performance of the OAD device was compared between devices on Die A and Die B, and the performance of the non‐OAD device was confirmed by comparing devices on Die C and Die D. In Figure S4 (Supporting Information), the average *μ* of Die A and Die B was found to be 0.45 and 0.40 cm^2^ V^−1^ s^−1^, respectively. The average on/off ratio between Die A and Die B showed a change of 1.8%, and the average *V_TH_* values were –9.58 and −9.02 V. The average *SS* for both dies were −2.13 and −2.12 V dec^−1^, showing little variation. Regarding the non‐OAD device, the transistor parameters between Die C and Die D also showed consistent values that aligned well with the tendency observed in the OAD device (Figure S5, Supporting Information).

**Figure 3:**
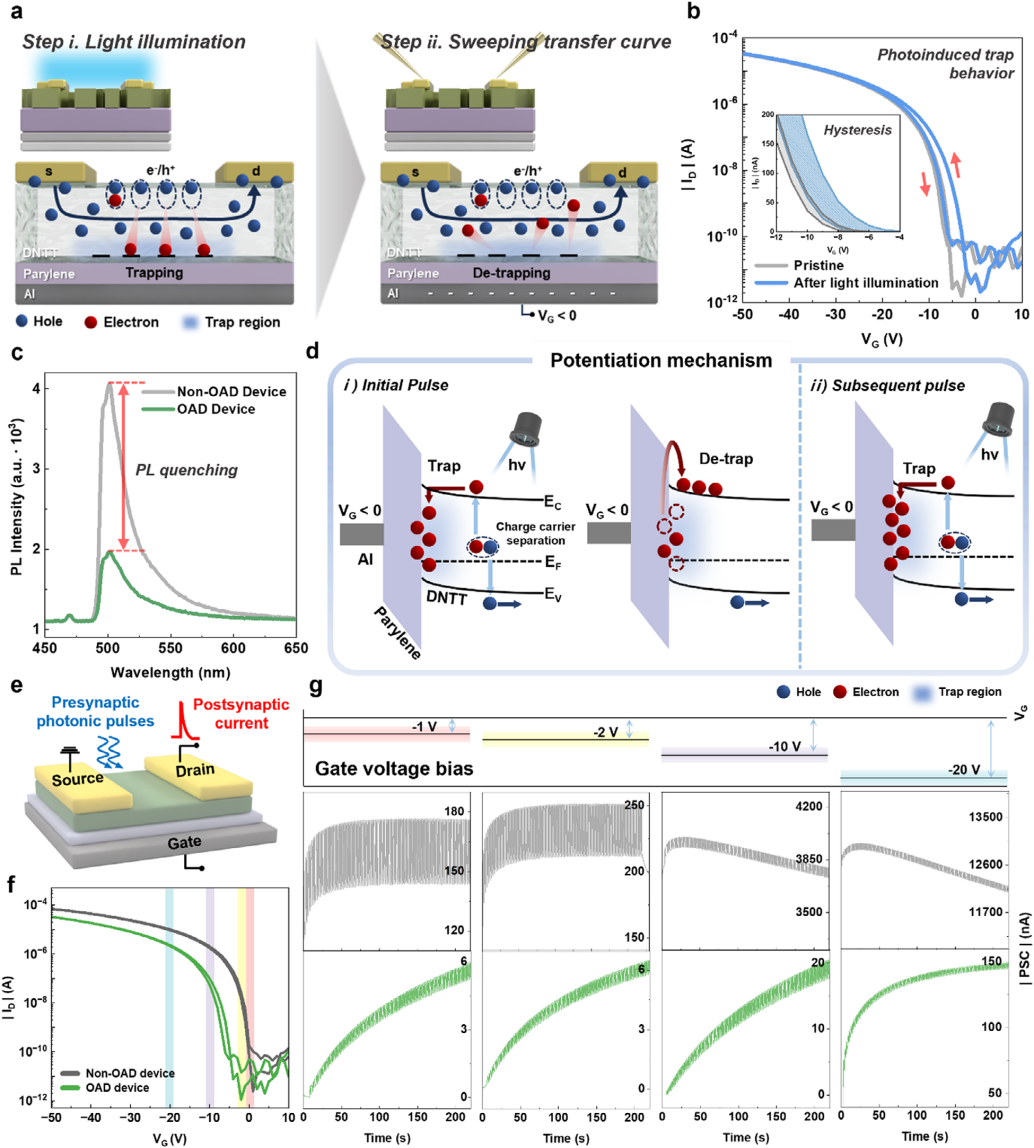
a) Photoinduced charge trapping mechanism and b) hysteresis in the transfer curve caused by photogating effect in the OAD device. c) PL spectra of the device with and without OAD. d) Mechanism of synaptic potentiation induced by photonic pulse. e) Schematic representation of a three‐terminal OAD device with synaptic characteristics. A presynaptic pulse applied to the channel generates PSC at the drain, depending on the *V_G_*. f) *V_G_* values used in synaptic measurements shown in the transfer curve. g) Synaptic characteristics at *V_G_* of −1 V, −2 V, −10 V, and −20 V for non‐OAD and OAD devices.

To investigate the trap effect by photonic stimulation, a 455 nm light source was utilized, showing strong absorption in both DNTT channels with and without OAD (Figure S6, Supporting Information). **Figure** **3**a describes the traps generation mechanism of the OAD device. In Step i, under a light with a wavelength of 455 nm, electron‐hole pairs were generated within DNTT, and electrons became trapped in the trap regions at the interface between DNTT with modified grains and Parylene. In Step ii, these trapped electrons are detrapped when a negative gate bias is applied after light illumination. As a result, hysteresis was observed as shown in Figure 3b, with a *V_TH_* shift of 2.07 V in response to the photogating effect, which returns to the pristine state due to the detrapping process induced by a negative gate bias. Conversely, when the non‐OAD device was measured using the same method, no hysteresis was observed (Figure S7, Supporting Information). This implies that the absence of OAD inhibited the formation of trap regions at the semiconductor‐gate dielectric interface, thereby suppressing the photogating effect. Furthermore, photoluminescence (PL) analysis was performed to investigate the formation of photoinduced trap regions of OAD and non‐OAD devices (Figure 3c). The PL intensity of the DNTT layer in the OAD device decreased compared to that of that in the non‐OAD device, indicating that photogenerated carriers were captured by trap sites and prevented from recombination. In other words, the modified DNTT morphology led to an increase in defect density, which acted as trap sites although there was no difference in light absorption between DNTT with and without OAD, as measured by utraviolet‐visible (UV–vis) spectroscopoy in Figure S6 (Supporting Information).

To shed light on the energy band structure, Tauc plots were obtained from the absorption spectra. The bandgaps of DNTT with and without OAD were measured to be 2.62 and 2.61 eV, respectively (Figure S8a, Supporting Information). A 455 nm light sufficiently exceeds the bandgaps of both devices, confirming that electron‐hole pairs can be generated upon exposure to this wavelength. Figure S8b (Supporting Information) shows ultraviolet photoelectron spectroscopy (UPS) spectra of DNTT. The Fermi levels of DNTT with and without OAD were −4.23 and −4.31 eV, and the valence band edges were 0.78 and 0.73 eV, respectively. As a result, the energy band diagram of DNTT depending on OAD was obtained with minimal difference (Figure S8c, Supporting Information). In the case of Au, a previous work was referenced.^[^ ^54^ ^]^ Despite the negligible differences in band structure and optical absorption, the OAD device exhibited a distinct hysteresis of 2.07 V under illumination, whereas the non‐OAD device showed no such behavior. This observation is further supported by the reduced PL intensity observed in the OAD device (Figure 3c), indicating that photogenerated carriers were trapped at the interface due to morphology changes induced by the OAD process.

Based on the results of the trap regions and energy band structure analyses, the photoinduced potentiation mechanisms of the OAD device were investigated (Figure 3d). When an initial light pulse is applied, electron‐hole pairs are generated and separated into the conduction and valence bands. Holes migrate through the channel while electrons become trapped in localized regions, causing a positive shift in *V_TH_* and an increase in current. In the absence of photonic stimulation, applying a negative gate bias releases trapped electrons, shifting *V_TH_* negatively and slightly reducing current. If light is reapplied before complete electron detrapping, additional electrons become trapped, gradually increasing the electron population in trap regions. This cyclic trap‐detrap process amplifies the photogating effect and progressively enhances current, effectively replicating the LTP behavior of synapses. In contrast to the potentiation behavior induced by photonic stimulation, synaptic depression was achieved through a *V_G_*‐driven detrapping process (Figure S9, Supporting Information). After electrons were trapped at the interface between the OAD‐modified DNTT grains and the Parylene dielectric, sequential application of *V_G_* pulses (−10 and −10.5 V, 1 s each) under dark conditions gradually released the trapped charges. As the pulses were repeatedly applied, the number of trapped electrons gradually decreased, weakening the gating effect on the channel caused by the trapped electrons, which led to a shift of *V_TH_* in the negative direction. As a result, the synaptic response gradually diminished, emulating long‐term depression (LTD) behavior.

As illustrated in Figure 3e, the artificial synaptic device comprises a three‐terminal transistor that uses photonic pulses with 0.27 mW cm^−2^ as stimulation. For the purpose of adjusting the synaptic weight, various *V_G_* values were applied as a supplementary input in Figure 3f. We adjusted the *V_G_* to −1, −2, −10, and −20 V to the device, categorizing *V_G_* into three regimes: Regime i at –1 and –2 V (subthreshold regime), Regime ii at –10 V (threshold regime), and Regime iii at –20 V (completely turned on regime). As a result, no synaptic behavior was observed in the non‐OAD device. In contrast, the OAD device showed potentiation with a gradual increase in current across all *V_G_* ranges in accordance with the previously discussed mechanism (Figure 3g). Normalized synaptic characteristics according to *V_G_* values are presented in Figure S10a (Supporting Information), with the most significant change in synaptic weight at *V_G_* = −10 V. Specifically, synaptic weight changes were quantified as the ratio of *W*_n_/*W*_1_, where *W*_n_ and *W*_1_ represent the PSCs at the nth and first pulses, respectively. The largest synaptic weight changes were observed at a *V_G_* of −10 V, with the most pronounced changes occurring at all of the 10th, 20th, 50th, and 100th photonic pulses, maintaining remarkable linearity of EPSC through to the 100th photonic pulse (Figure S10b, Supporting Information). At *V_G_* = –10 V, the *W*_100_/*W*_1_ value reached 182.13 A A^−1^, which represents an ≈8419.7% enhancement compared to the value of 1.09 A·A^−1^ at *V_G_* = –20 V, where the smallest change in synaptic weight was observed. This result clearly demonstrates that synaptic weights in our device can be precisely modulated by up to 85‐fold solely through *V_G_* control. These variations of synaptic weights depending on *V_G_* arise from the varying photogating effects in each regime. For effective electron trapping, photogenerated electron‐hole pairs should be separated, allowing electrons to be trapped at the DNTT/Parylene interface. However, in the absence of a formed channel and with restricted carrier transfer, the recombination of electron‐hole pairs becomes more probable than when the channel is formed (Regime i). With the increase of *V_G_* and the channel formation (Regime ii), photogating effect is enhanced indicating more significant synaptic weight change. Electron‐hole pairs become more easily separated, facilitating hole escape to the drain through the channel and promoting electron trapping. In the completely turned on regime, the photogating effect diminishes due to full formation of the channel and relatively strong negative *V_G_*. Thus, *V_G_*‐tunable synaptic characteristics were achieved through the OAD, leveraging different photogating effects that depend on *V_G_*. These outcomes facilitate efficient weight adjustment in neural network learning and enable a precise emulation of biological synapses.

Synaptic behavior was also investigated under photonic stimulation at wavelengths of 530 and 660 nm, with a constant intensity of 0.27 mW cm^−2^ (Figure S11, Supporting Information). Despite the emergence of potentiation, a pronounced lack of changes in synaptic weights was discerned compared to the potentiation curve under 455 nm photonic pulses. To clarify, the 530 and 660 nm lights were challenging to stimulate the OAD device in contrast to the obvious synaptic behavior induced by 455 nm photonic stimulation, as DNTT showed minimal light absorption to green and red light (Figure S6, Supporting Information). Accordingly, we have extensively characterized the synaptic mimicry of the OAD device using 455 nm photonic stimulation at *V_G_* of −10 V.

**Figure 4:**
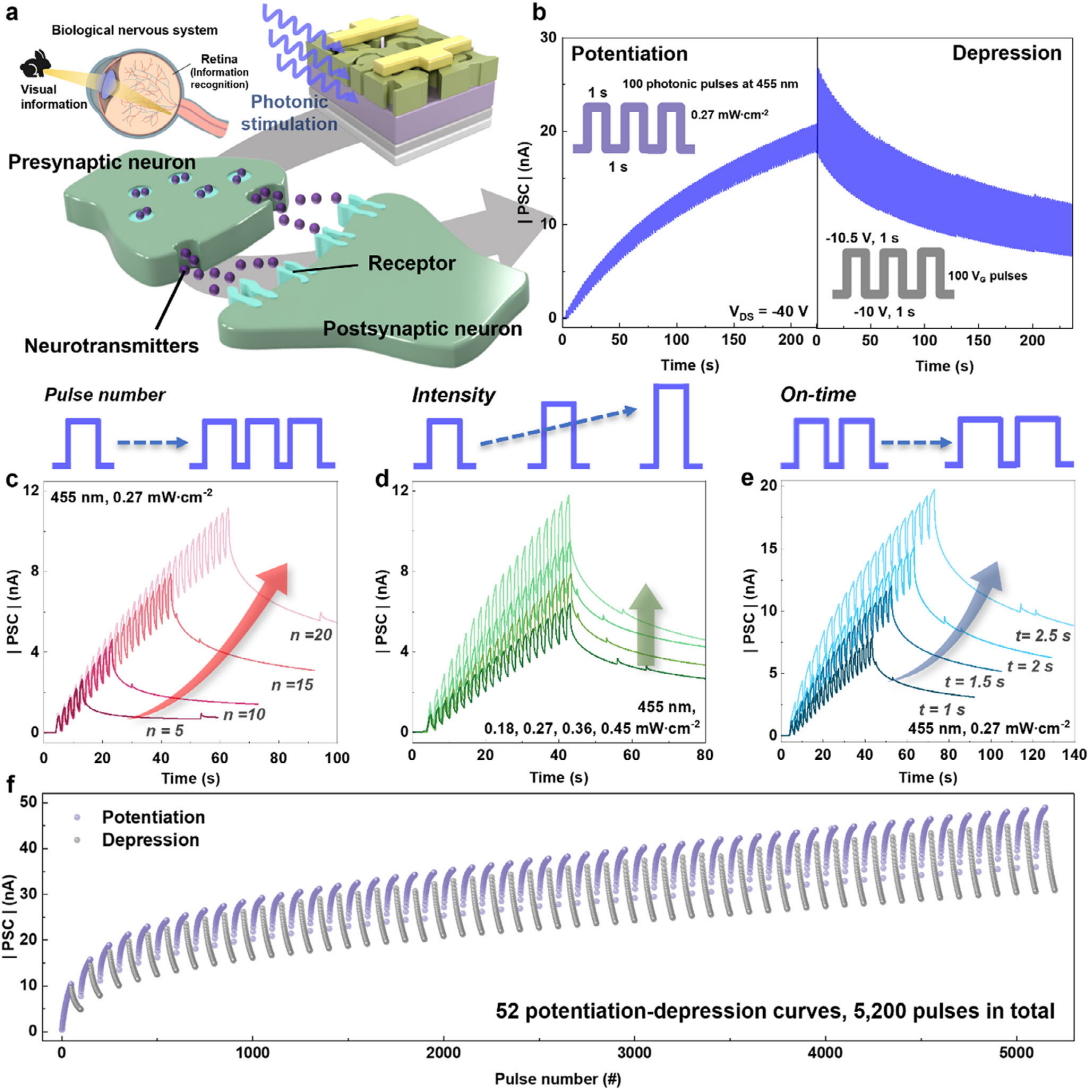
a) Schematic illustration of neural connectivity through synapses activated by stimuli in the biological nervous system. b) Potentiation and Depression curve of the OAD device with presynaptic pulses applied. Synaptic plasticity of the OAD device: Dependence on c) the number of pulses, d) light intensity, and e) light on‐time. f) Potentiation and depression curves measured repeatedly for 52 cycles. Both potentiation and depression were produced by using 50 photonic and *V_G_* pulses, respectively.

In the human body, sensory organs like the eyes, nose, and skin detect stimuli and relay them to the central nervous system, which then directs motor responses. Neurons transmit these signals electrically, while synapses, the junctions between neurons, process, integrate, and store information via neurotransmitters (**Figure** **4**a).^[^ ^55^, ^56^, ^57^ ^]^ We successfully mimicked biological synapses using two types of presynaptic inputs (i.e. photonic and *V_G_* pulses) to generate action potentials. The resulting *I_D_* serves as the postsynaptic output. Figure S12 (Supporting Information) details optoelectronic synaptic behavior of the OAD device.

Figure 4b illustrates the potentiation‐depression curve of the OAD device under specific conditions (*V_G_* = −10 V and *V_DS_* = −40 V). Upon applying 100 photonic pulses with an intensity of 0.27 mW cm^−2^ and a wavelength of 455 nm, the postsynaptic current (PSC) gradually increased from 0.09 to 17 nA. Depression was achieved by applying 100 *V_G_* pulses, alternating between −10 V for 1 s and −10.5 V for 1 s, which reduced the PSC to 6.79 nA (Figure S13, Supporting Information). The traps induced by photonic stimulation are alleviated through subsequent electrical stimulation. Additionally, the potentiation‐depression curve was implemented at *V_G_* = −10 V and *V_DS_* = −2 V, although the change in synaptic weights was significantly smaller compared to those observed at *V_DS_* = −40 V (Figure S14, Supporting Information). To further understand the effect of *V*_*DS*_ on synaptic characteristics by photonic stimulation, we calculated the synaptic weight change. As shown in Figure S15 (Supporting Information), *W*_100_/*W*_1_ at *V_DS_* = −40 V was 182 A·A^−1^ for potentiation. In contrast, the values of 17 A·A^−1^ were presented at *V_DS_* = −2 V, indicating a much smaller change in synaptic weight. These findings suggest that the OAD device exhibits *V_DS_*‐dependent synaptic characteristics. To investigate the effect of OAD‐induced morphological changes on synaptic behavior, we conducted a control experiment based on film thickness. As shown in Figure S16 (Supporting Information), the DNTT film deposited via OAD exhibited a thickness of ≈40 nm, as confirmed by AFM analysis, which is thinner than that of the non‐OAD device (≈60 nm). Accordingly, we fabricated an additional non‐OAD device with the same thickness (40 nm) to match that of the OAD device and analyzed its synaptic characteristics. As shown in Figure S17 (Supporting Information), the non‐OAD device with a 40 nm thickness did not exhibit synaptic behavior, and although a slight increase in current was observed at *V_G_* = –10 V, the synaptic weight *W*_100_/*W*_1_ was only 1.09 A A^−1^ significantly lower than 182.13 A A^−1^ observed in the OAD device. These results experimentally confirm that the synaptic characteristics are induced by morphology modulation and associated interfacial trap formation through the OAD process.

Next, we demonstrated the transition from short‐term memory (STM) to long‐term memory (LTM) by controlling the photonic stimulation. The PSC progressively increased with the pulse number (5, 10, 15, and 20), intensity (0.18, 0.27, 0.36, and 0.45 mW cm^−2^), and on‐time (1, 1.5, 2, and 2.5 s) (Figure 4c–e). Minor fluctuations observed in the PSC after photonic stimulation did not affect the stable maintenance of synaptic weight following the end of stimulation. These fluctuations are considered to be negligible noise that did not influence LTM behavior and the interpretation of synaptic characteristics. In other words, the synaptic weights could be dynamically adjusted by varying the stimulation conditions. Furthermore, DNTT is known for its excellent environmental stability, including resistance to oxidation, thermal degradation,^[^ ^3^, ^58^ ^]^ and bias stress. To evaluate the stability of the synaptic characteristics in the OAD device using this property of DNTT, a cycle test was performed using continuously repeated potentiation‐depression curves, leveraging the stability of DNTT. As shown in Figure 4f, potentiation‐depression curves were reliably maintained throughout 52 cycles, corresponding to a total of 5,200 pulses, demonstrating robust durability against external stimuli (i.e. photonic and *V_G_* pulses). Additionally, synaptic strength in biological systems may progressively increase or decrease depending on stimulus conditions during repeated potentiation and depression processes. These 52 potentiation‐depression curves, which exhibited the increase in PSC during cycles, mimicked the biological behavior of gradually strengthening synaptic connections through repeated potentiation and depression processes by stimuli.

Although the morphology engineering of DNTT led to a degradation of transistor performance, the OAD device validated successful synaptic plasticity, with the capability to modulate synaptic weights, as evidenced by the potentiation‐depression curve and the transition from STM to LTM. As another observation, the remarkable stability observed over 52 repeated potentiation‐depression cycles highlights the potential of our optoelectronic synaptic device for practical applications in fields such as robotics, autonomous systems, and neurorehabilitation.

To further advance the applicability for image recognition and classification of the OAD device, the Modified National Institute of Standards and Technology (MNIST) handwritten digit simulation was performed. As shown in Figure S18a (Supporting Information), the artificial neural network (ANN) consists of 784 input neurons, 10 output neurons, and a hidden layer of 256 neurons in between. The potentiation‐depression data of the OAD device at *V_DS_* = −40 V were integrated into the crossbar array for the final output, where model optimization was conducted (Figure S18b, Supporting Information). Further details of the simulation are provided in the experimental section.

Figure S19a (Supporting Information) shows the handwritten digit images with true and predicted labels, classified using the OAD device‐based ANN. The true and predicted labels correspond to the values of the input image and the output values, respectively. In the confusion matrix, most handwritten digit images were accurately categorized, with the true labels matching the predicted labels, resulting in a maximum accuracy of 92.6% during 100 training epochs (Figure S19b,c, Supporting Information). Meanwhile, the potentiation‐depression curves at *V_G_* = −1, −2, and −20 V were implemented under the same stimulation conditions and also evaluated by MNIST handwritten digit simulation (Figure S20a, Supporting Information). The accuracies were similar to that at *V_G_* = −10 V, exceeding 91% at *V_G_* = −1, −2, and −20 V (Figure S20b, Supporting Information). Thus, the reliable synaptic performance of the OAD device at different *V_G_* values was validated through the successful classification in Figure S20c–e (Supporting Information).

**Figure 5:**
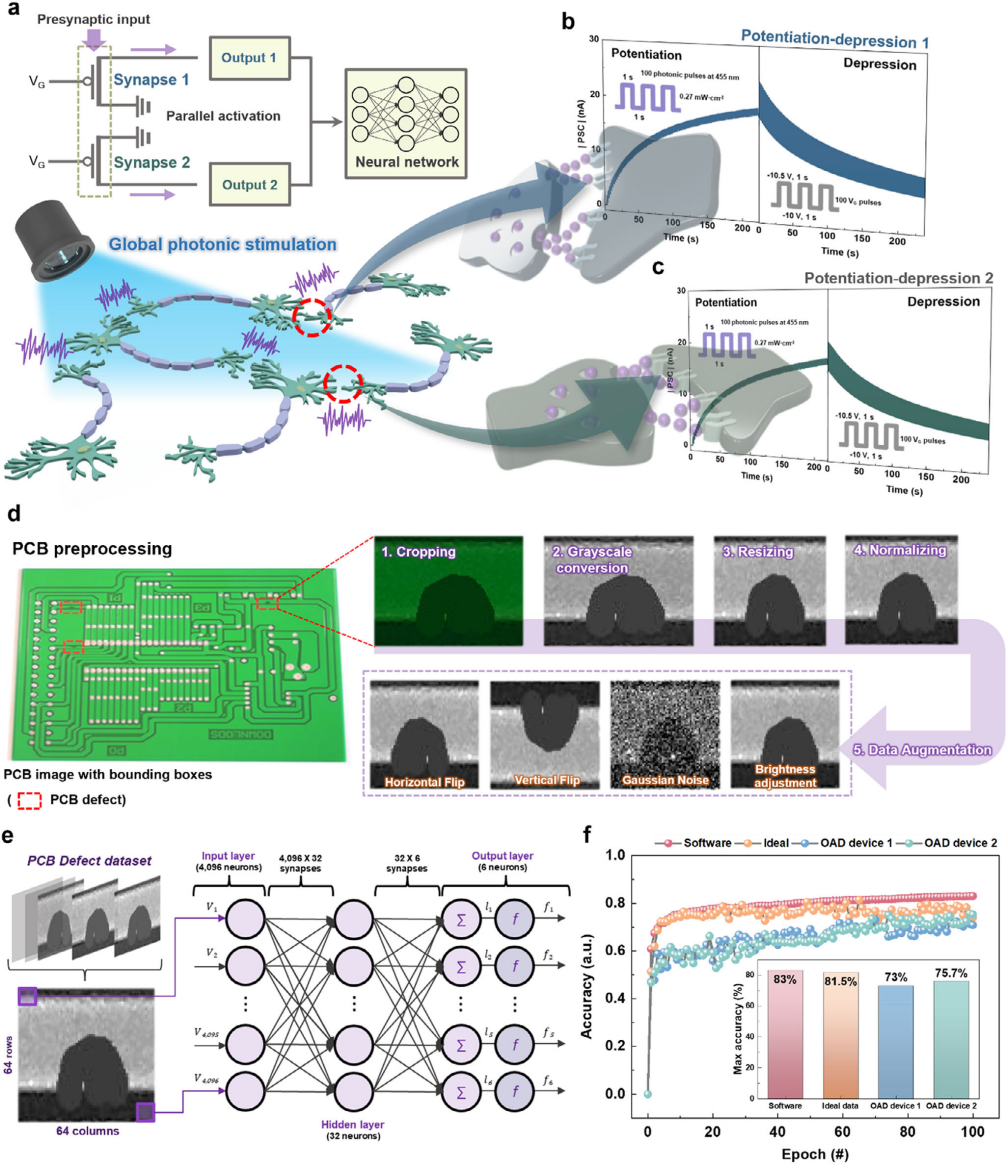
a) Schematic illustration of synapses simultaneously stimulated by global photonic pulses. The circuit describes parallel activation of two independent OAD devices for the application to ANN. Parallel potentiation‐depression curves of b) OAD device 1 and c) OAD device 2. Schematic illustration of d) preprocessing of data for PCB defect inspection and e) OAD device based‐ANN. f) Training accuracy for PCB defect with respect to different epoch from ANN using OAD device 1, OAD device 2, software, and ideal data.

Our results demonstrate the ability of the OAD device to enable parallel operation, a crucial feature for efficient data processing. Powered by AI and machine vision, industries generate and analyze vast amounts of data. Parallel computing optimizes resource utilization, enabling faster and more energy‐efficient processing, which is an essential aspect of neuromorphic engineering.^[^ ^59^, ^60^, ^61^ ^]^ We present the OAD device, which is designed to facilitate parallel data processing by leveraging the unique property of photonic stimulation. The OAD devices were simultaneously stimulated through photonic synchronization, leading to the generation of parallel potentiation‐depression curves. **Figure** **5**a schematically illustrates how to achieve parallel activation of synaptic transistors. Two OAD devices were subjected to the presynaptic input (i.e. 100 photonic pulses) from a single light source in the same condition as the previous measurement (*V_G_* = −10 V, *V_DS_* = −40 V, and 455 nm light intensity of 0.27 mW cm^−2^). PSCs were independently generated at the drain of each transistor. Figure 5b,c displays two potentiation‐depression curves. After 100 photonic pulses, the PSC levels reached 17.57 and 16.17 nA, respectively (Figure S21, Supporting Information), closely matching the previous result of 17 nA in Figure S13 (Supporting Information). This confirms the successful parallel generation of uniform potentiation‐depression curves by the consistency of photonic signals.

Numerous studies have already presented DNTT transistor arrays in various applications utilizing vacuum deposition systems.^[^ ^62^, ^63^, ^64^, ^65^, ^66^ ^]^ In this situation, the OAD device with OAD‐engineered DNTT, which enables parallel operation, offers a tremendous advantage when fabricated at array scale, facilitating efficient and large‐scale neuromorphic computing. As another neuromorphic application using the photonic‐stimulation, we apply our device to PCB defect detection. PCBs are crucial components in almost all electronic devices, providing the physical interconnection between circuits. As electronic devices and semiconductor chips become increasingly integrated, the demand for advanced PCB defect detection and inspection grows. Machine vision‐based methods have largely replaced traditional approaches, like manual inspection, due to their faster speeds and reduced risk of visual fatigue.^[^ ^67^, ^68^ ^]^ In this context, we performed PCB defect detection by incorporating two parallel potentiation‐depression curves (Figure S21, Supporting Information) into an ANN. Figure 5d,e outlines the inspection process using the OAD device. First, the defect regions in the PCB image are cropped using a bounding box. Then, grayscale conversion, resizing, and normalization optimize the data for the ANN model. Data augmentation techniques (horizontal flip, vertical flip, Gaussian noise, and brightness adjustment) ensure training diversity. The preprocessed data is fed into an OAD device‐based ANN with 4,096 input neurons, 6 output neurons, and a hidden layer of 32 neurons, enabling classification for PCB defect detection. The experimental section provides further details on the inspection process. Figure 5e shows the PCB defect inspection results using two OAD devices stimulated in parallel. The maximum accuracy reached 73% and 75.7% after 100 training epochs. It is a value that is close to the accuracy of 81.5% and 83%, which are the expected maximum accuracy using artificially generated ideal potentiation‐depression data and software, and it is almost identical to the OAD device simulation results. This demonstrates that OAD devices, activated simultaneously by photonic stimulation, can classify PCB defects in parallel (inset of Figure 5e).

We have conducted comparative reviews of various studies on synaptic devices and PCB defect detection to illustrate device‐specific attributes and network‐level performance of the OAD device. In Table S1 (Supporting Information), recently reported organic synaptic transistors, including the OAD device, were examined.^[^ ^69^, ^70^, ^71^, ^72^, ^73^ ^]^ The OAD device demonstrated competitive synaptic characteristics, including synaptic plasticity, dynamic range (>182), 100 conductance states, and cycle durability (>52). Notably, it achieved 92.6% accuracy in the MNIST handwritten digit simulation, placing it at the higher end of the reported performance range (75%–93%). Next, Table S2 (Supporting Information) presents an analysis of the recently reported models for PCB defect detection.^[^ ^68^, ^74^, ^75^, ^76^ ^]^ Previous studies have predominantly adopted software‐based convolutional neural network (CNN) models, whereas our work demonstrated a hardware‐based multilayer perception (MLP) model that directly incorporates the LTP/LTD properties of the synaptic device into the weight update process. Despite the relatively simpler structure of the MLP compared to CNN architecture, our approach to PCB defect inspection achieved a meaningful accuracy of 75.7%, which is close to the 81.5% accuracy obtained using a network model with ideal device parameters. In addition, although our accuracy is slightly lower than that of other studies shown in Table S2 (Supporting Information), this is attributed to the inherent advantage of CNN models in processing image data compared to MLP models. Consequently, our organic synaptic transistor possessed outstanding synaptic characteristics and showcased the potential for hardware ANN‐based PCB defect inspection, rather than a conventional software‐based system.

## 3 Conclusion

In this work, we engineered a *V_G_*‐tunable organic synaptic transistor by utilizing OAD of DNTT to generate photoinduced trap behavior. Notable differences in grain morphology and electrical properties were observed between OAD and non‐OAD devices, particularly with the formation of trap regions at the DNTT‐Parylene interface. We demonstrated successful optoelectronic synaptic behavior through a series of plasticity and durability tests. Synaptic behavior was observed across a range of *V_G_* values, highlighting the device's suitability for efficient weight adjustment in ANN learning. The proposed OAD devices show promise for photonic stimulation‐based neuromorphic systems. The ability to generate uniform potentiation‐depression curves under parallel stimulation conditions underscores their potential for high‐throughput classification tasks. In the proposed application to the PCB defect detection, OAD devices effectively identified defects, achieving performance levels that are commensurate with those observed in ideal data simulations, making them highly suitable for advanced machine vision applications in electronics quality control and inspection.

## 4 Experimental Section

### Fabrication of OAD and Non‐OAD Device

2.5 cm × 2.5 cm glass substrates were cleaned by ultrasonication in acetone and isopropanol (IPA) for 15 min each, followed by rinsing with deionized (DI) water to remove residues. Nitrogen (N₂) blowing was then performed to dry the substrates. Al gate was deposited via thermal evaporation (the deposition rate of 1–3 Å s^−1^). Parylene, used as the gate dielectric, was coated through Parylene coater. For the OAD device, OAD with the deposition angle of 80° was applied to modify the DNTT morphology while DNTT of non‐OAD device was deposited using a conventional thermal evaporation method (the deposition rate was 0.2–0.3 Å s^−1^). Then, Au source/drain electrode were deposited via thermal evaporation (the deposition rate was 0.9–1 Å s^−1^). All semiconductors and electrodes were patterned using a shadow mask.

### Characterization of OAD and Non‐OAD Devices

DNTT morphology was analyzed using SEM (SEM, S‐4700, Hitachi, Tokyo, Japan). The absorption spectrum of each material was measured by UV–vis spectroscopy (UV–vis, PerkinElmer Lambda 750, The Commonwealth of Massachusetts, USA) and the energy bandgaps were extracted from Tauc plots. UPS was performed to determine the secondary cut off and the valence band edge of DNTT (UPS, AXIS SUPRA, Kratos. Inc., California, USA). To understand the charge trapping behavior, PL was analyzed by spectrofluorometer (PL, Nanolog, Horiba, Kyoto, Japan) with Xe lamp as a light source. Additionally, a charge coupled device (CCD) detector with liquid nitrogen incorporated was used to enhance signal‐to noise ratio. For measuring the electrical characteristic, including current‐voltage (*I*‐*V)* or current‐time (*I*‐*T)* behavior, a probe station and a Keithley 4200A‐SCS were used under ambient conditions.

### MNIST Handwritten Digit Simulation

To assess the performance of the OAD device in terms of image recognition and classification, MNIST handwritten digit simulation was conducted using the ANN. The ANN was composed of input (784 neurons), hidden (256 neurons), and output (10 neurons) layers, with synaptic matrices between each layer where the potentiation‐depression data of the OAD device were applied. The inputs were fed into the simulation model by converting 2D handwritten images of 28 × 28 scale to one dimension. To ensure the stability of the simulation model, the potentiation‐depression data from the OAD device were normalized and then converted to one dimension. In the simulation, synaptic weights were expressed as the difference in conductance values of two equivalent synapses, and the final output was determined by repeating forward propagation, backward propagation, and weight updates. During backward propagation, the predicted values from the forward propagation calculated by the input data were compared to the actual data, generating gradients that were used to adjust the weights according to their sign, thereby optimizing the model performance.

### PCB Defect Simulation

For the PCB defect simulation, the dataset was sourced from an open‐access repository, containing PCBs with six types of defects: missing hole, mouse bite, open circuit, short circuit, spur track, and spurious copper pattern, alongside defect‐free PCBs. The dataset underwent five preprocessing steps: box bounding, cropping, grayscale conversion, resizing, normalization, and data augmentation. Box bounding involves determining the coordinates of a rectangle that outlines the defect within the PCB image. Cropping then extracts the image based on these coordinates. Resizing adjusts the image to a resolution of 64 × 64 pixels. Normalization was performed to ensure the stability of the simulation model. Data augmentation, including horizontal and vertical flipping, brightness adjustment, and the introduction of Gaussian noise, were employed to prevent overfitting and improve the model generalization capability in the presence of limited data for each class. This enhanced data diversity. The ANN was composed of input (4,096 neurons), hidden (32 neurons), and output (6 neurons) layers. As with the MNIST handwritten digit simulation, the process of forward propagation, backward propagation, and weight updates was repeated and then the final outputs are determined.

## Conflict of Interest

The authors declare no conflict of interest.

## Supporting information



Supporting Information

## Data Availability

The data that support the findings of this study are available from the corresponding author upon reasonable request.
