# Supplementary material for: Organic Synaptic Transistors and Printed Circuit Board Defect Inspection with Photonic Stimulation: A Novel Approach Using Oblique Angle Deposition
Source: Small. 2025 May 7;21(25):2501997. doi: 10.1002/smll.202501997 (PMC12199130; doi:10.1002/smll.202501997)
Supplement: Supplementary file 1 — Supporting Information [file SMLL-21-2501997-s001.docx]

**Supporting Information**

**Organic Synaptic Transistors and Printed Circuit Board Defect Inspection with Photonic Stimulation: A Novel Approach Using Oblique Angle Deposition**

Gyeongho Lee, Yeo Eun Kim, Hyeonjung Kim, Han-Koo Lee, Jae Yeon Park, Seyong Oh^*^, and Hocheon Yoo^*^

This PDF file includes the following:

**Figure S1**. Transfer curves of 36 OAD devices in the dark state and under 455 nm light.

**Figure S2.** Transfer curves of 36 non-OAD devices in the dark state and under 455 nm light.

**Figure S3.** OM images of the measured 36 transistors from both the OAD and non-OAD devices, respectively.

**Figure S4.** Extracted electrical parameters from 18 transfer curves of Die A and Die B, respectively: a) mobility, b) on/off ratio, c) *V_TH_*, and d) *SS* statistics, including mean values and standard deviations.

**Figure S5.** Extracted electrical parameters from 18 transfer curves of Die C and Die D, respectively: a) mobility, b) on/off ratio, c) *V_TH_*, and d) *SS* statistics, including mean values and standard deviations.

**Figure S6.** UV-Vis absorption spectra of DNTT with and without OAD.

**Figure S7.** Photoinduced charge trapping mechanism and absence of hysteresis in the transfer curve due to the lack of photogating effect in the non-OAD device.

**Figure S8.** a) Tauc plots of DNTT with and without OAD, obtained from UV-Vis absorption spectra. b) Secondary cut-off region and valence band edge region of DNTT with and without OAD measured through UPS. c) Energy band diagrams of DNTT with OAD, DNTT without OAD, and Au.

**Figure S9.** Mechanism of synaptic depression induced by negative *V_G_* pulse.

**Figure S10.** a) Normalized *V_G_*-dependent PSC of the OAD device (*V_DS_* = $-$40 V). b) Synaptic weight changes at the 10th, 20th, 50th, and 100th photonic pulses.

**Figure S11.** EPSC behaviors of the OAD device under 455 nm, 530 nm, and 660 nm photonic pulses (*V_DS_* = $-$40 V and *V_G_* = $-$10 V).

**Figure S12.** Optoelectronic synapse operation of the OAD device.

**Figure S13**. PSC values extracted from the potentiation-depression curve of the OAD device at *V_DS_* = $-$40 V.

**Figure S14.** a) Potentiation-depression curve of the OAD device device at *V_DS_* = $-$2 V. b) PSC values extracted from the potentiation-depression curve of the OAD device at *V_DS_* = $-$2 V.

**Figure S15.** Comparison of synaptic weight changes in the OAD device at *V_DS_* = $-$2 V and $-$40 V under the same *V_G_* of $-$10 V.

**Figure S16.** a) Thickness of the DNTT layer deposited on the non-OAD device. b) Thickness of the DNTT layer deposited on the OAD device. c) Thickness of the non-OAD thin-film device deposited to match the thickness of the OAD device.

**Figure S17.** a) Synaptic characteristics of the non-OAD device with a 40 nm thickness under various *V_G_* values. b) Synaptic characteristics of the OAD device under various *V_G_* values.

**Figure S18.** a) Schematic diagram of a three-layer ANN for learning MNIST handwritten digit images. b) Synaptic weight is the difference in conductivity between two synaptic devices.

**Figure S19.** MNIST simulation results from the OAD device at *V_G_* = $-$10 V. a) Handwritten digit images with true and predicted labels, classified using the OAD device-based ANN. b) Confusion matrix for ANN, where rows represent the desired output digits, and columns represent the predicted output numbers. Correct classifications are located on the diagonal of the table. c) Training accuracy for handwritten image recognition with respect to different epochs.

**Figure S20**. a) Potentiation-Depression curves and b) training accuracy for handwritten image recognition with respect to different epochs at *V_G_* = $-$1 V, $-$2 V, and -20 V. c) Confusion matrix at *V_G_* = $-$1 V, d) $-$2 V, and e) $-$20 V.

**Figure S21.** PSC values extracted from the potentiation-Depression curve of a) OAD device 1 and b) OAD device 2, respectively.

**Table S1.** Comparison of organic synaptic device characteristics.

**Table S2.** Comparison of defect detection accuracy with recent software models.


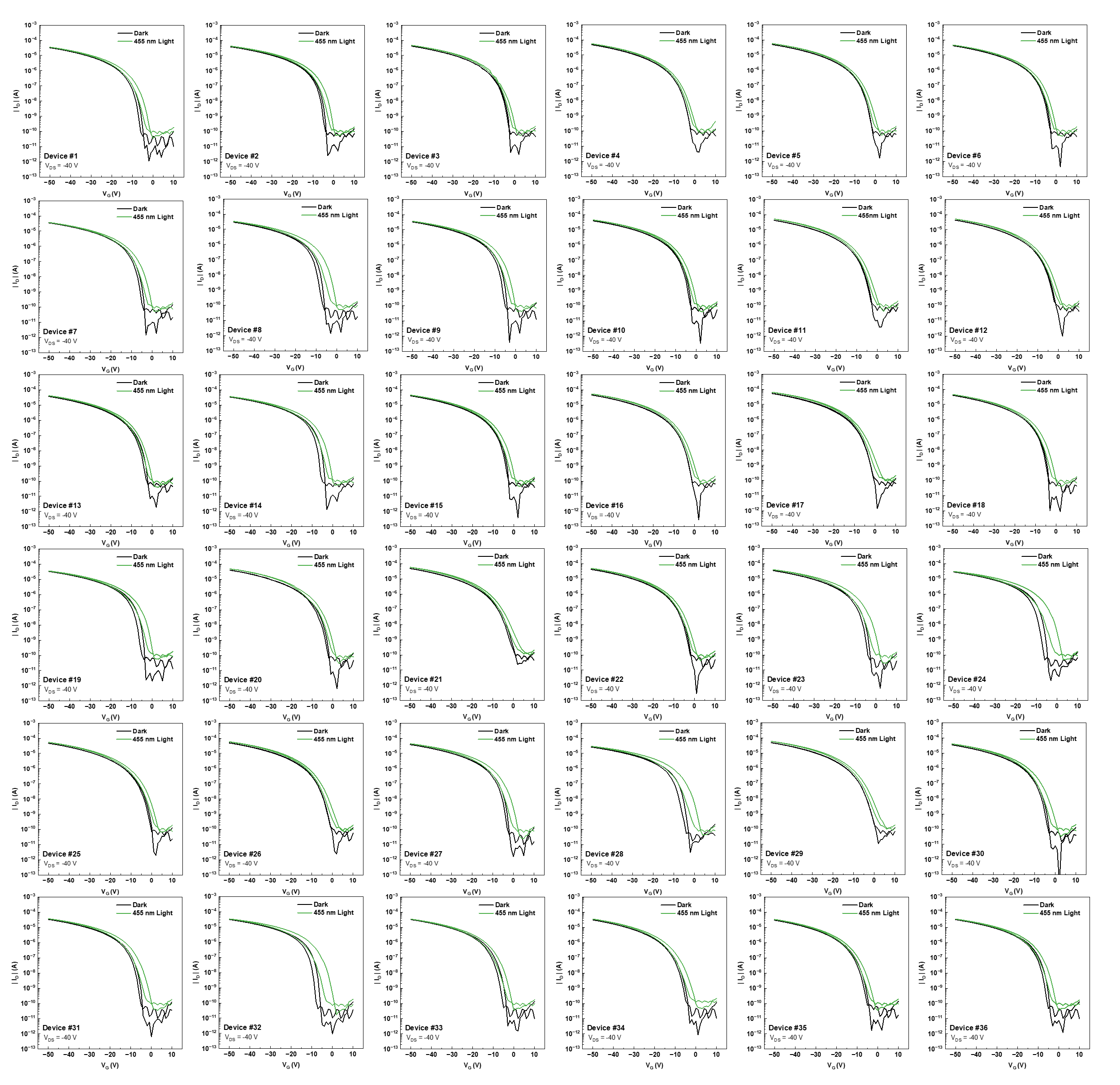


**Figure S1.** Transfer curves of 36 OAD devices in the dark state and under 455 nm light.


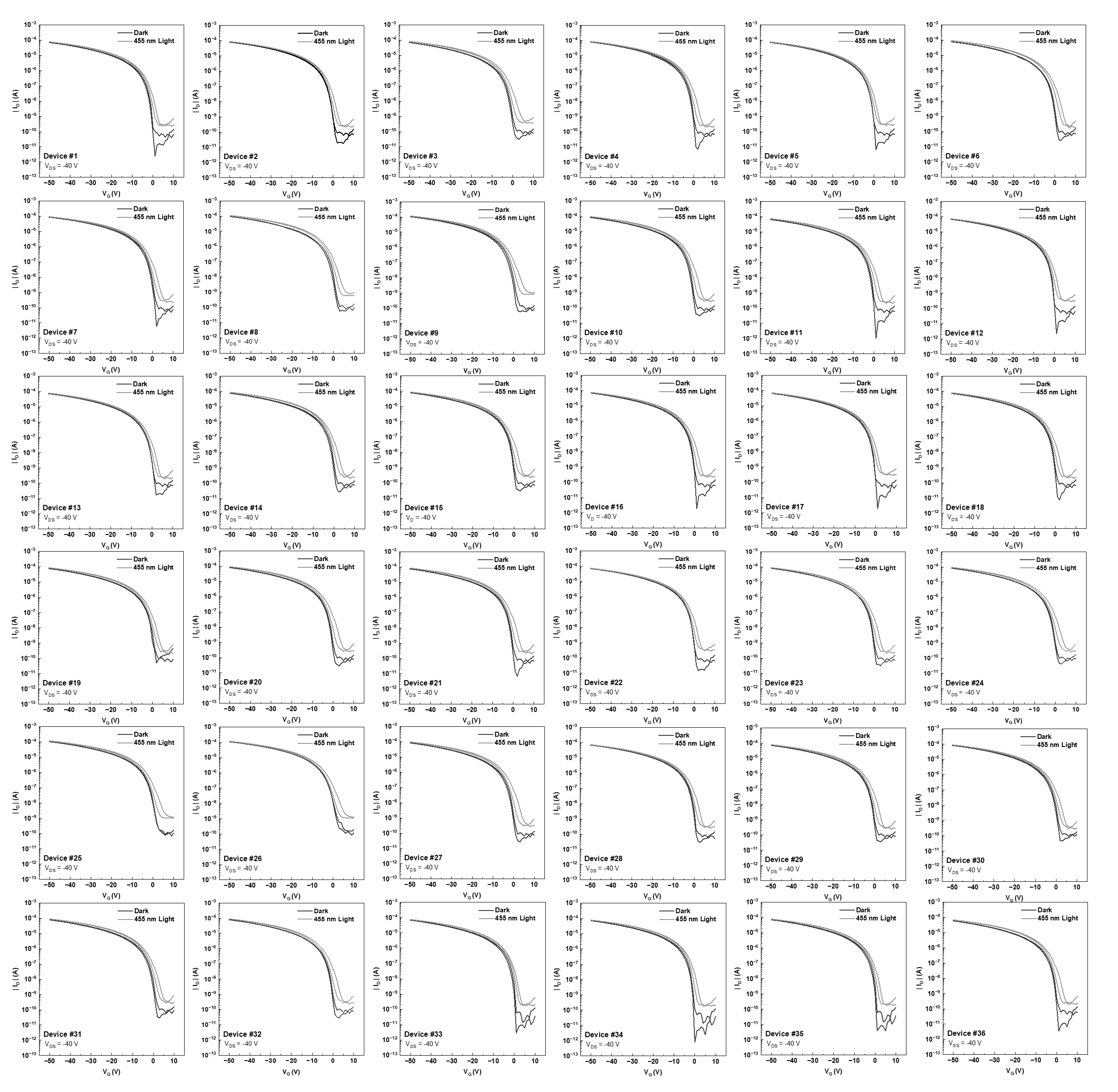


**Figure S2.** Transfer curves of 36 non-OAD devices in the dark state and under 455 nm light.


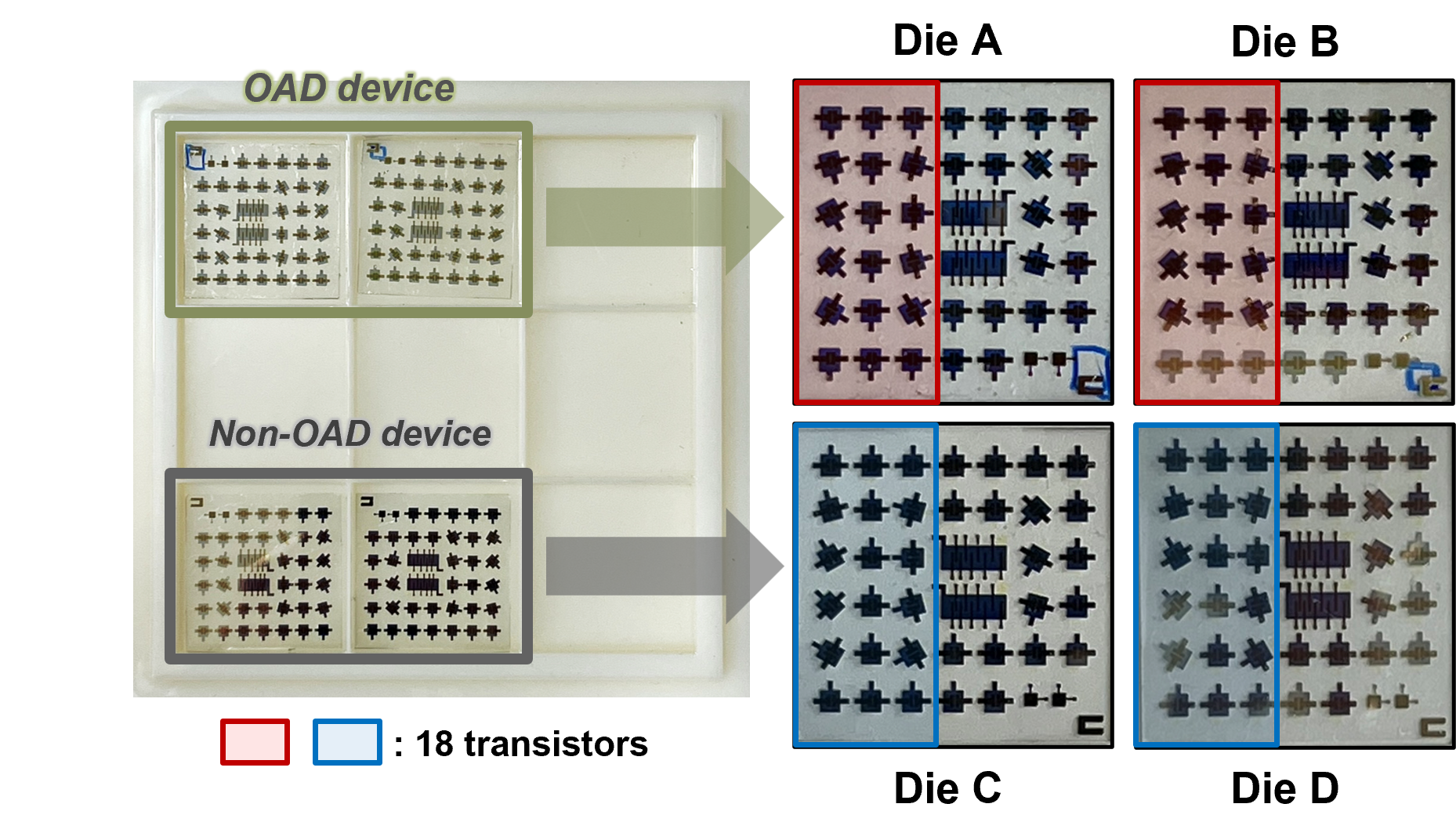


**Figure S3.** OM images of the measured 36 transistors from both the OAD and non-OAD devices, respectively.


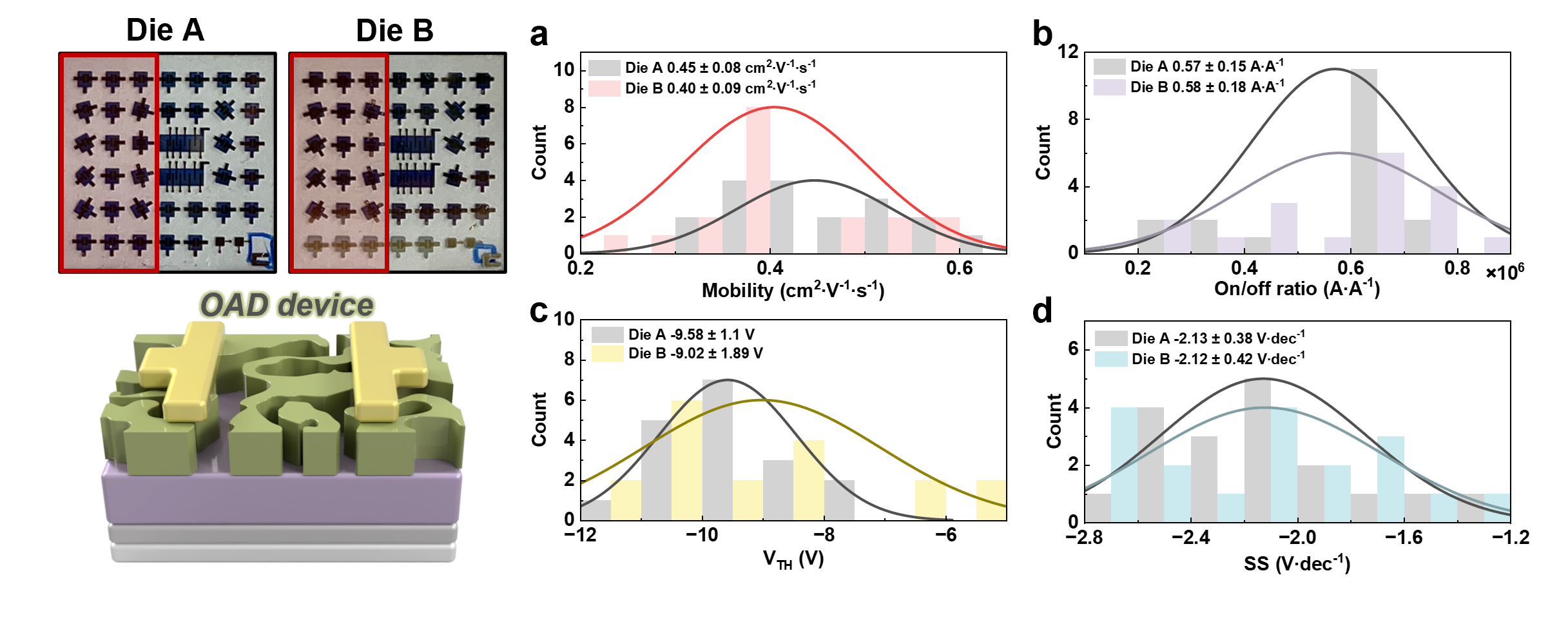


**Figure S4.** Extracted electrical parameters from 18 transfer curves of Die A and Die B, respectively: a) mobility, b) on/off ratio, c) *V_TH_*, and d) *SS* statistics, including mean values and standard deviations.


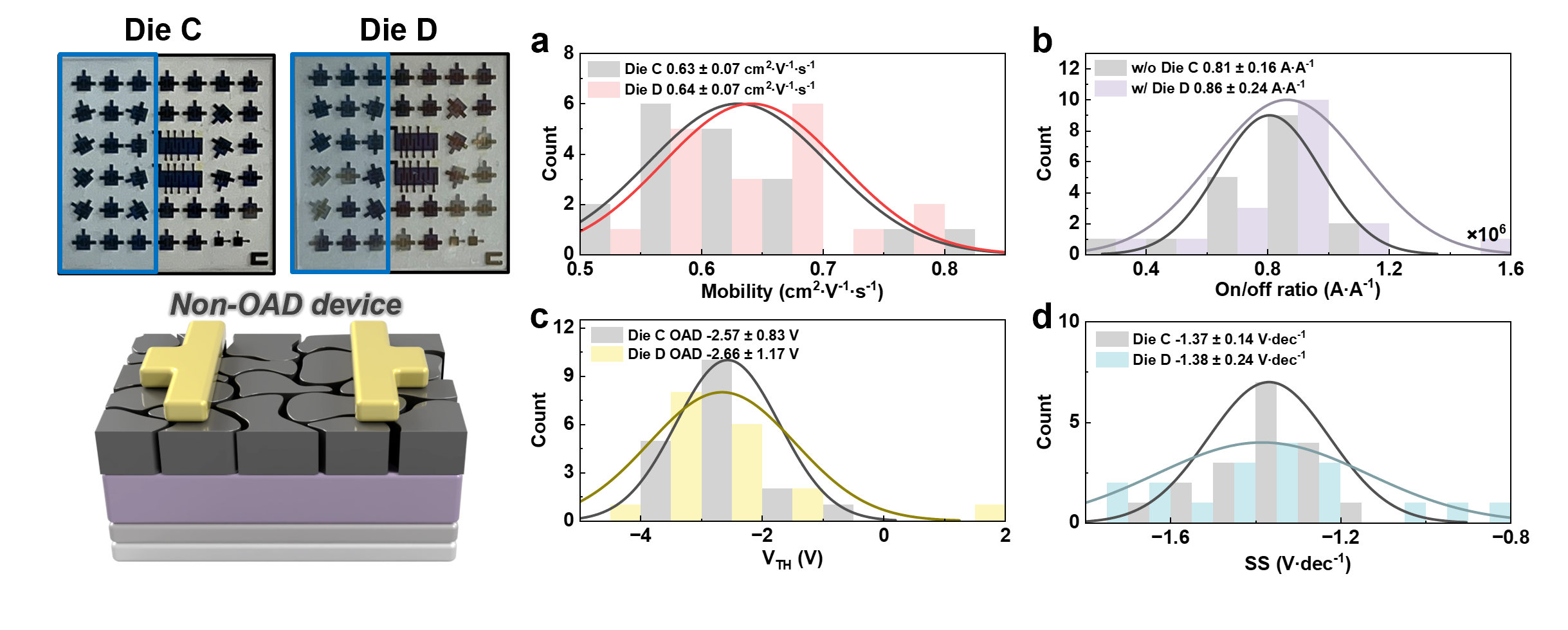


**Figure S5.** Extracted electrical parameters from 18 transfer curves of Die C and Die D, respectively: a) mobility, b) on/off ratio, c) *V_TH_*, and d) *SS* statistics, including mean values and standard deviations.


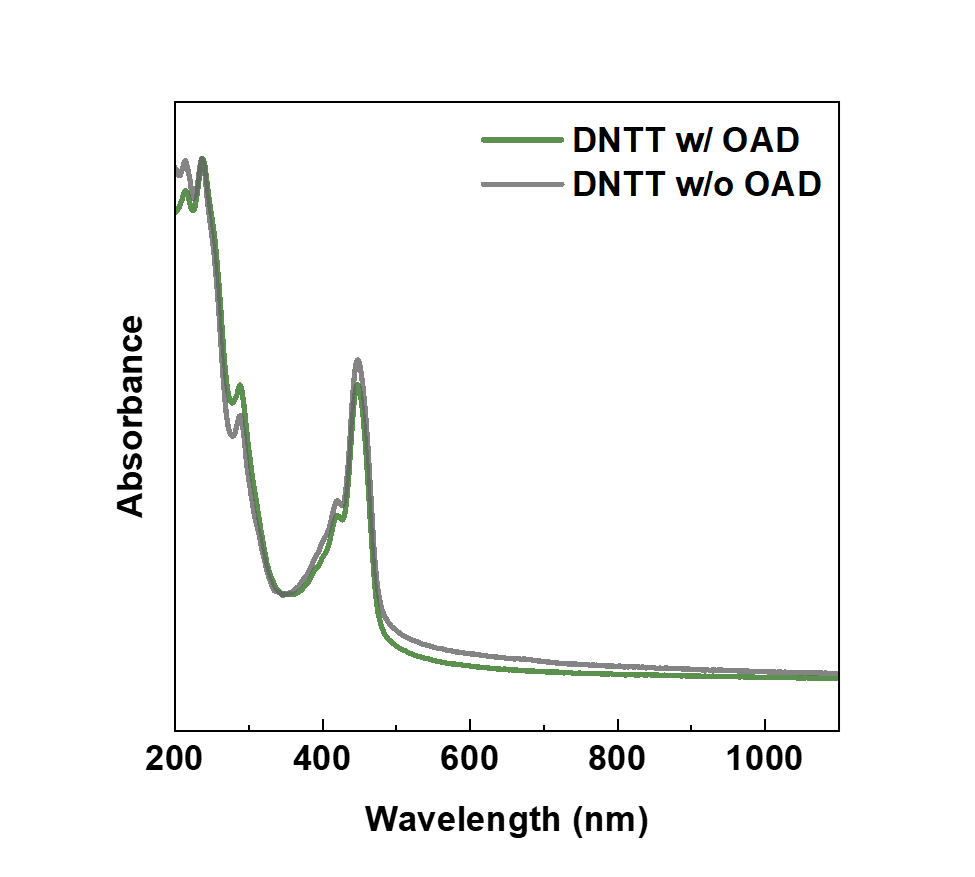


**Figure S6.** UV-Vis absorption spectra of DNTT with and without OAD.


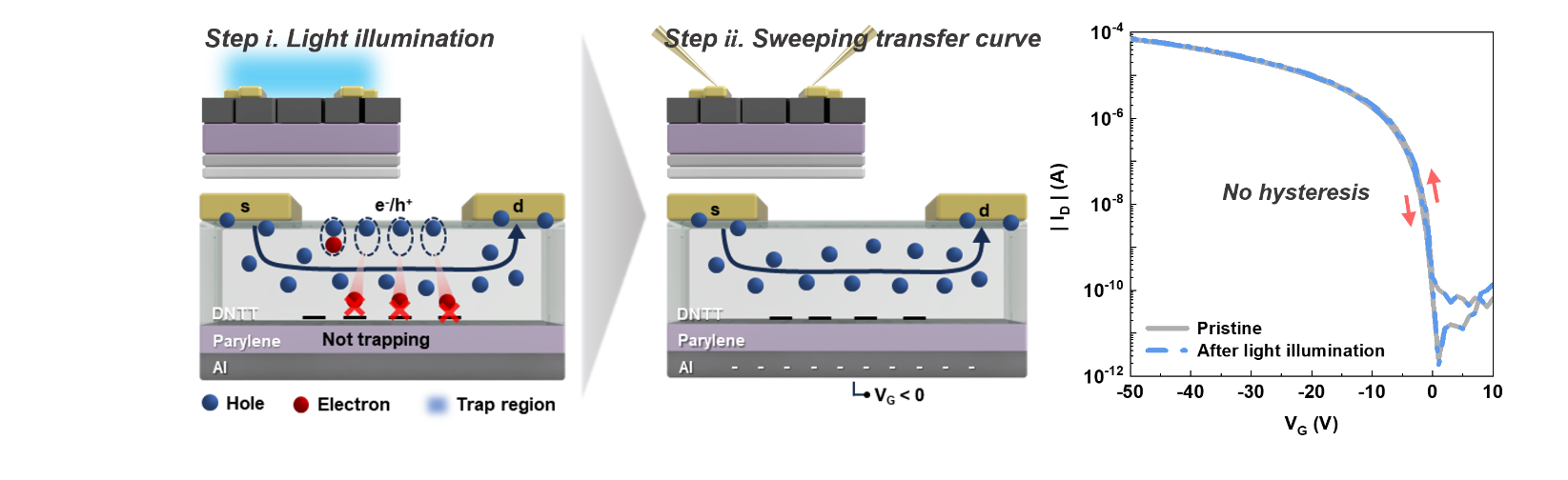


**Figure S7.** Photoinduced charge trapping mechanism and absence of hysteresis in the transfer curve due to the lack of photogating effect in the non-OAD device.


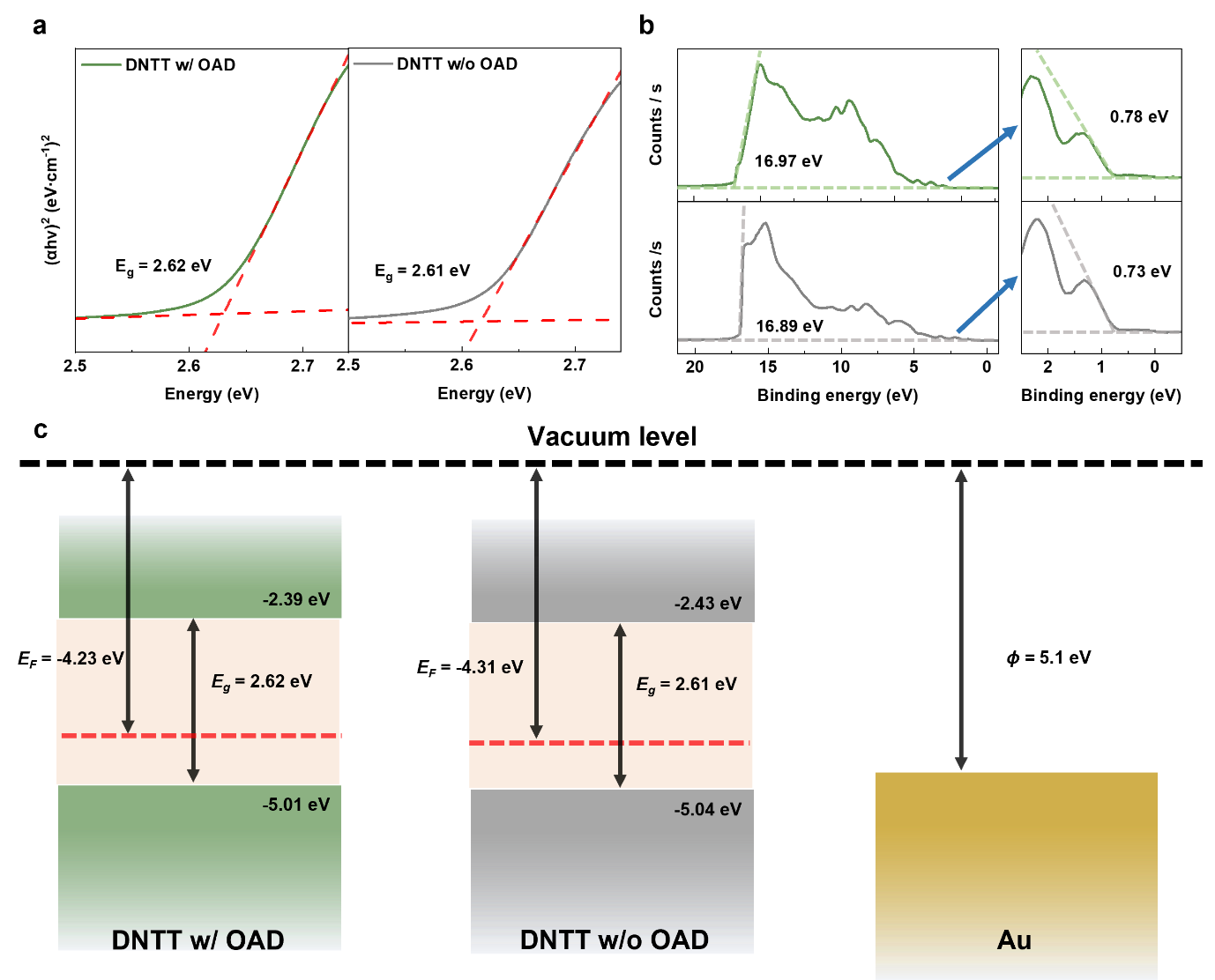


**Figure S8.** a) Tauc plots of DNTT with and without OAD, obtained from UV-Vis absorption spectra. b) Secondary cut-off region and valence band edge region of DNTT with and without OAD measured through UPS. c) Energy band diagrams of DNTT with OAD, DNTT without OAD, and Au.

**
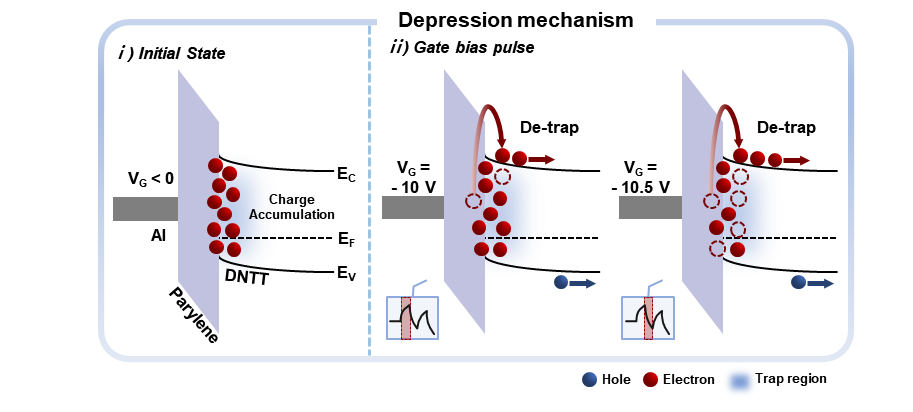
**

**Figure S9.** Mechanism of synaptic depression induced by negative *V_G_* pulse.


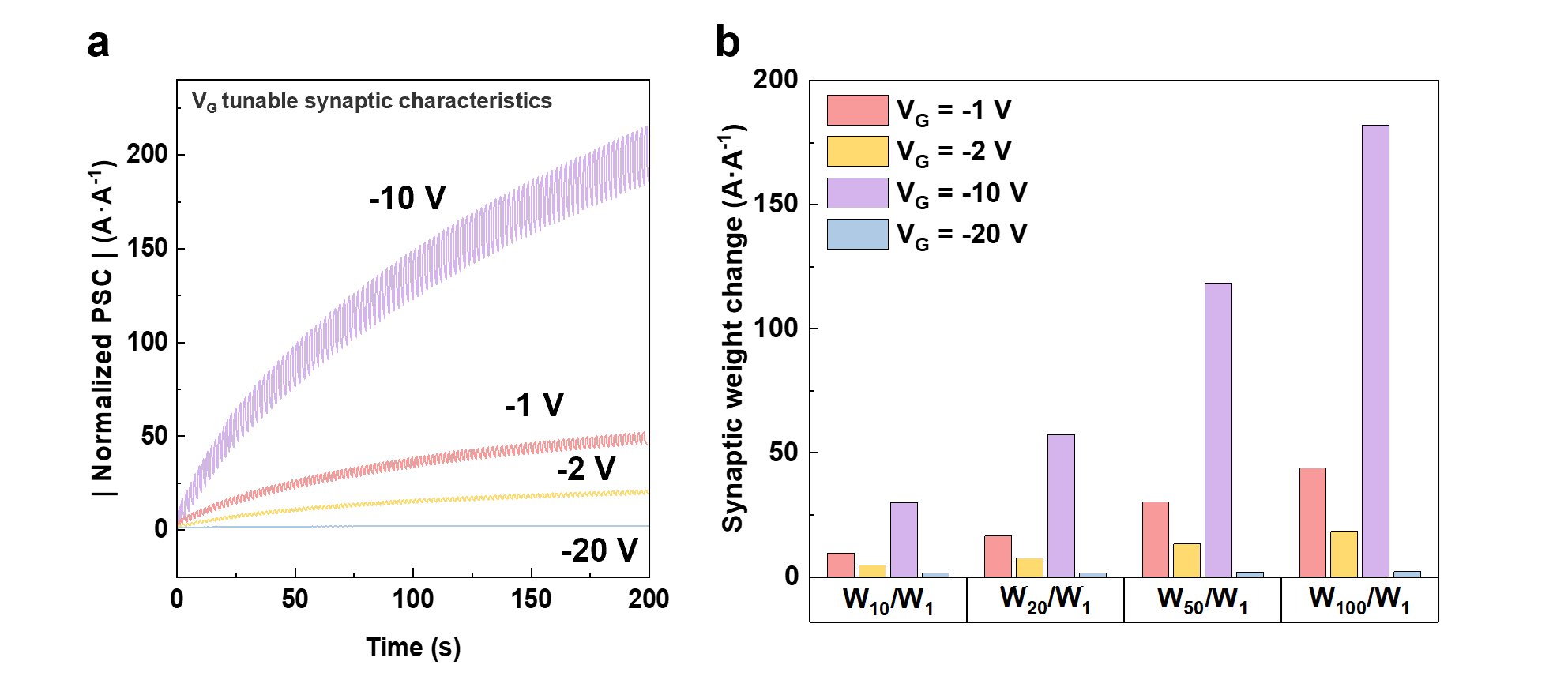


**Figure S10.** a) Normalized *V_G_*-dependent PSC of the OAD device (*V_DS_* = $-$40 V). b) Synaptic weight changes at the 10th, 20th, 50th, and 100th photonic pulses.


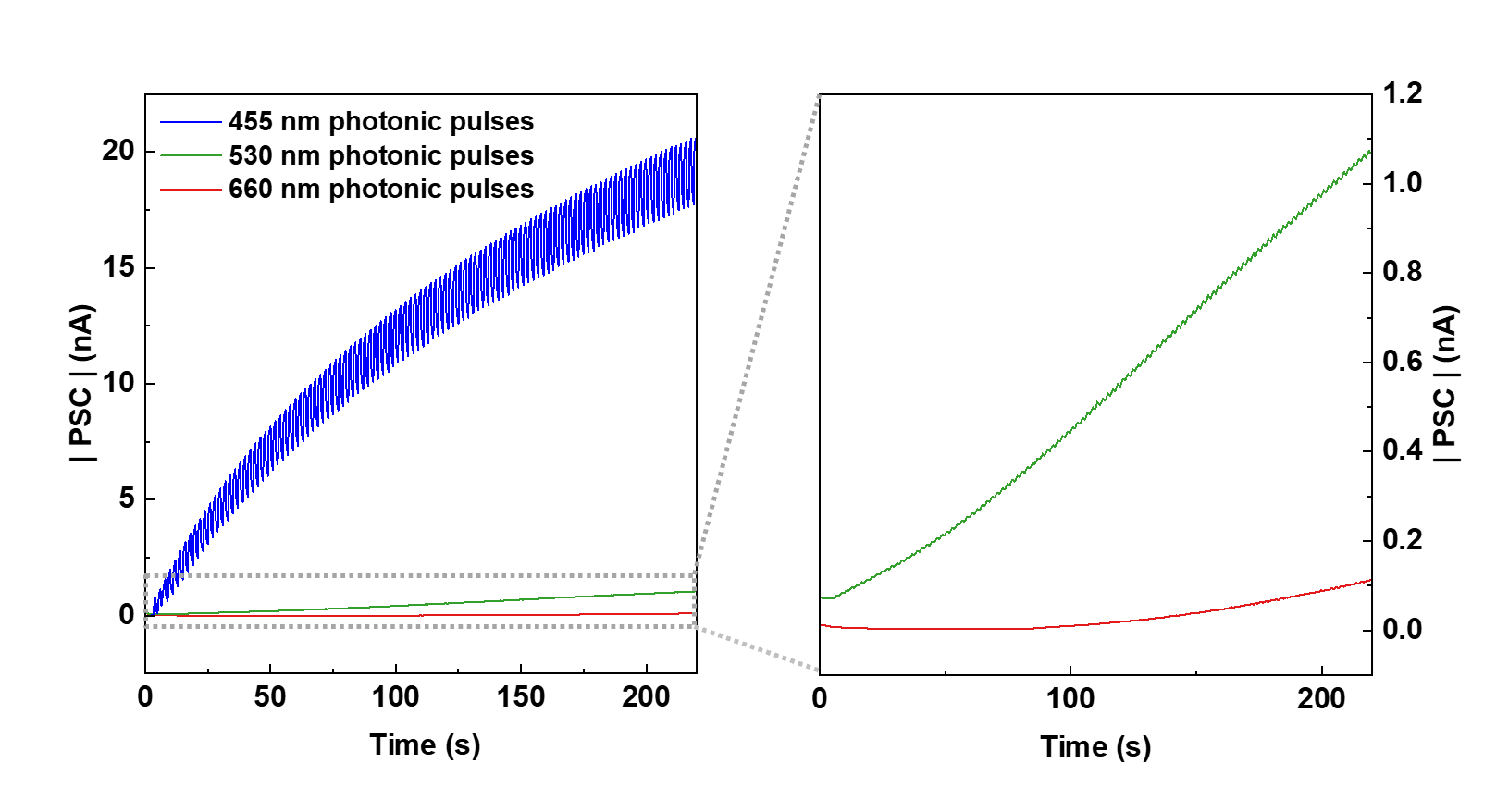


**Figure S11.** EPSC behaviors of the OAD device under 455 nm, 530 nm, and 660 nm photonic pulses (*V_DS_* = $-$40 V and *V_G_* = $-$10 V).


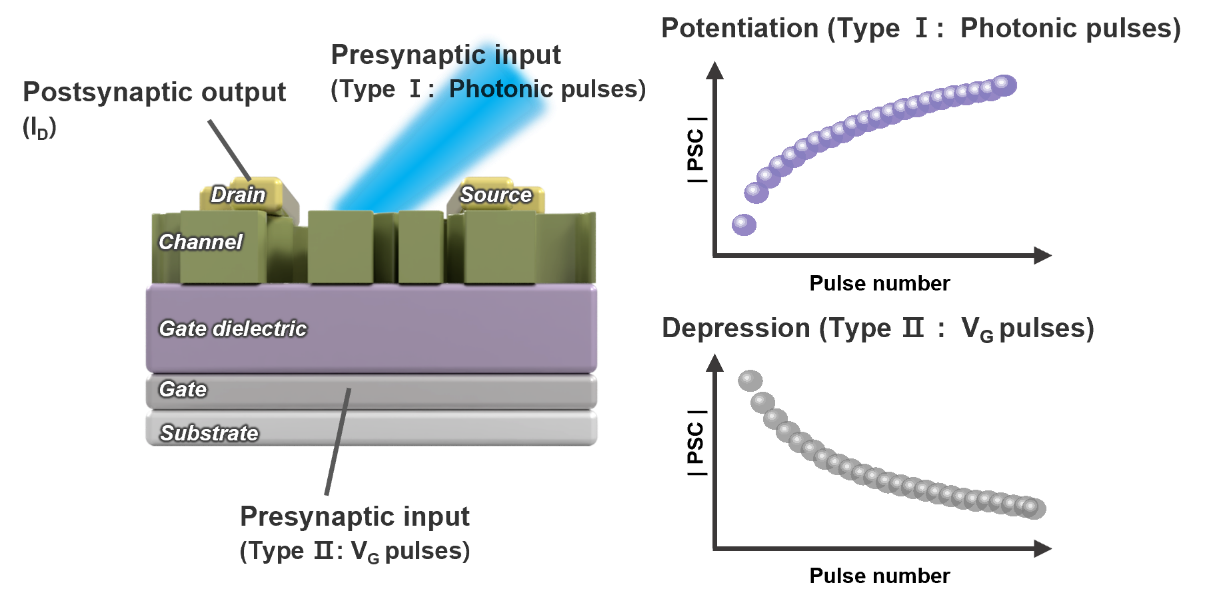


**Figure S12.** Optoelectronic synapse operation of the OAD device.


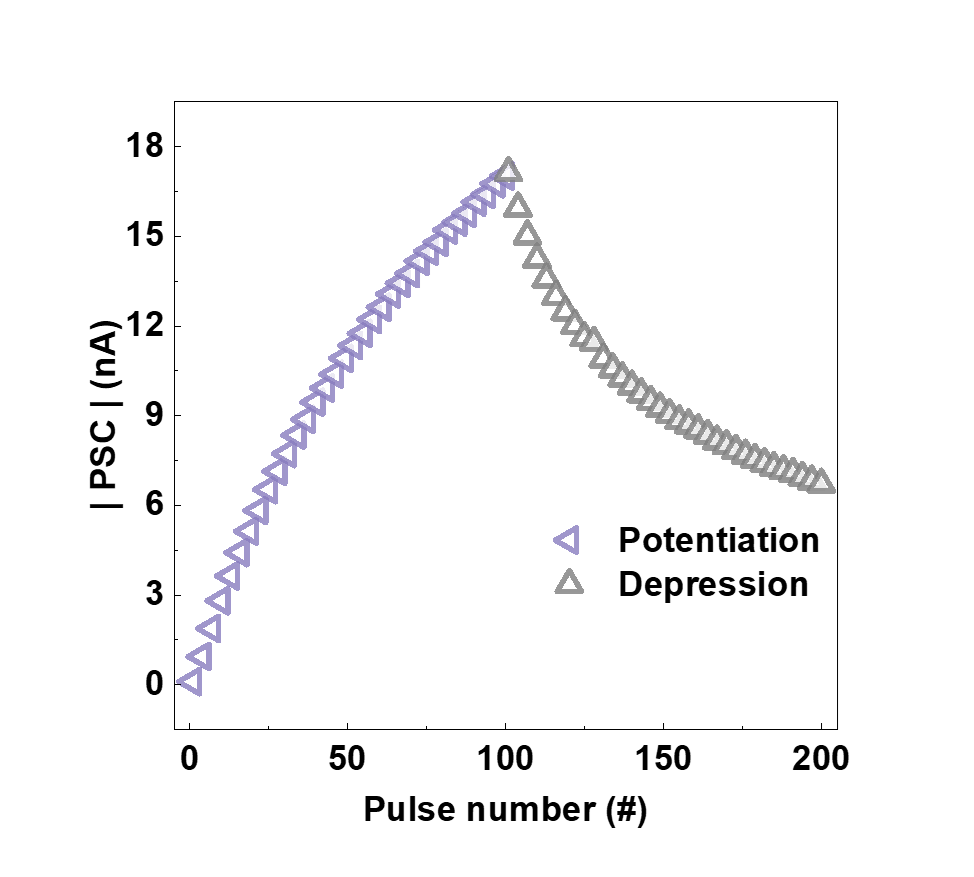


**Figure S13**. PSC values extracted from the potentiation-depression curve of the OAD device at *V_DS_* = $-$40 V.


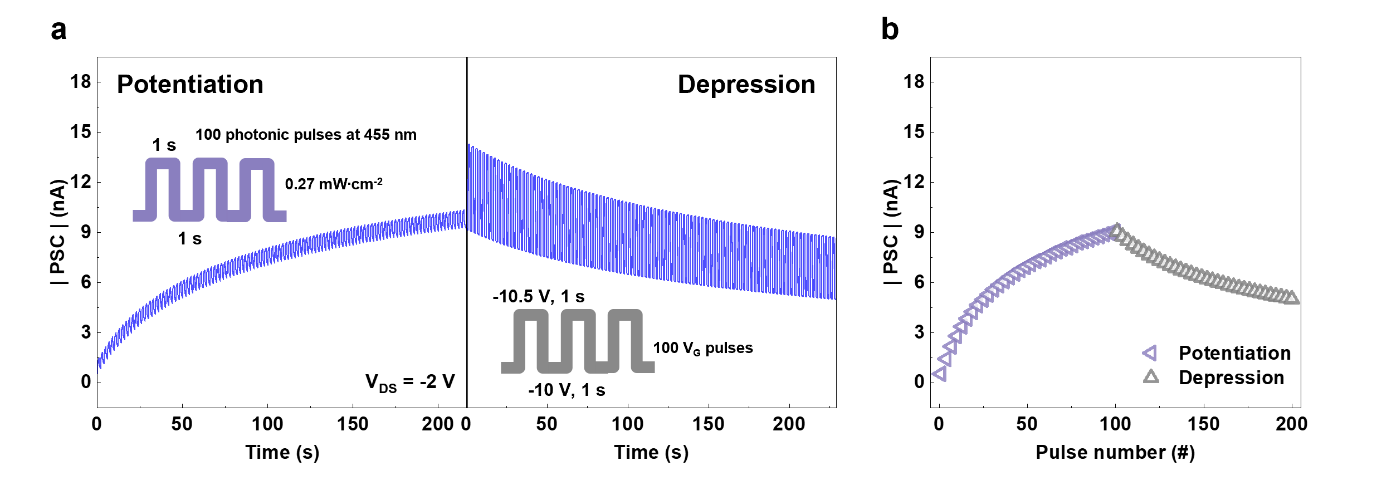


**Figure S14.** a) Potentiation-depression curve of the OAD device device at *V_DS_* = $-$2 V. b) PSC values extracted from the potentiation-depression curve of the OAD device at *V_DS_* = $-$2 V.


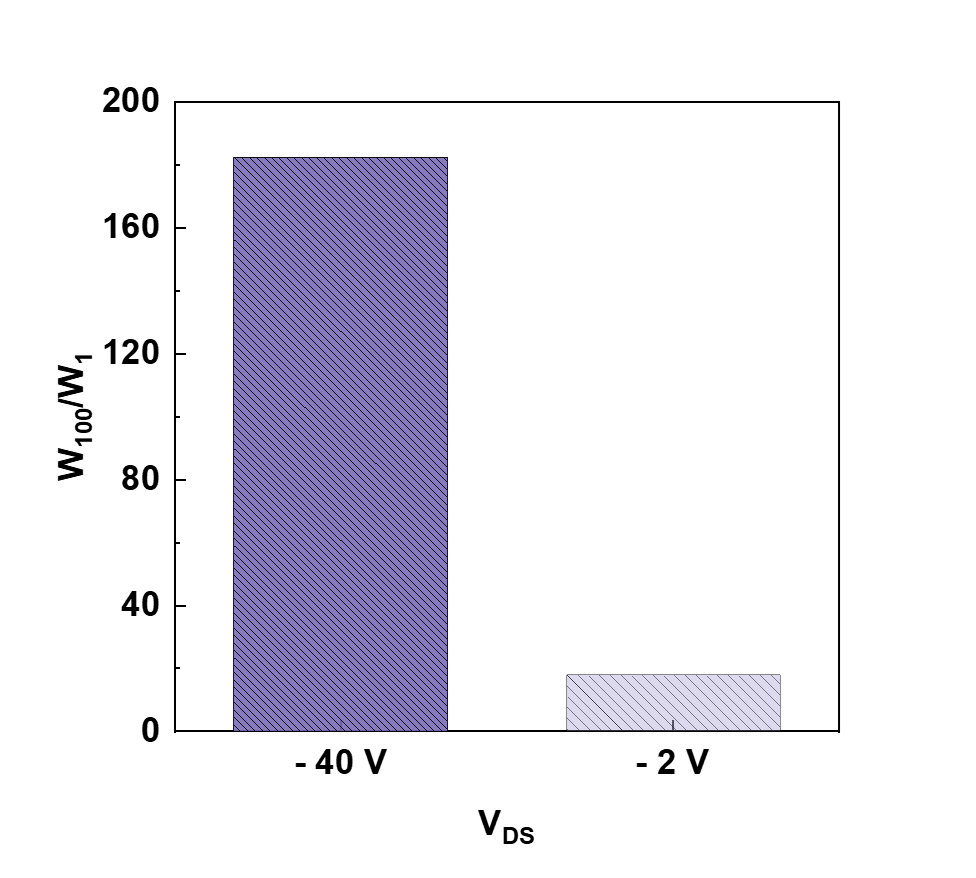


**Figure S15.** Comparison of synaptic weight changes in the OAD device at *V_DS_* = $-$2 V and $-$40 V under the same *V_G_* of $-$10 V.

**
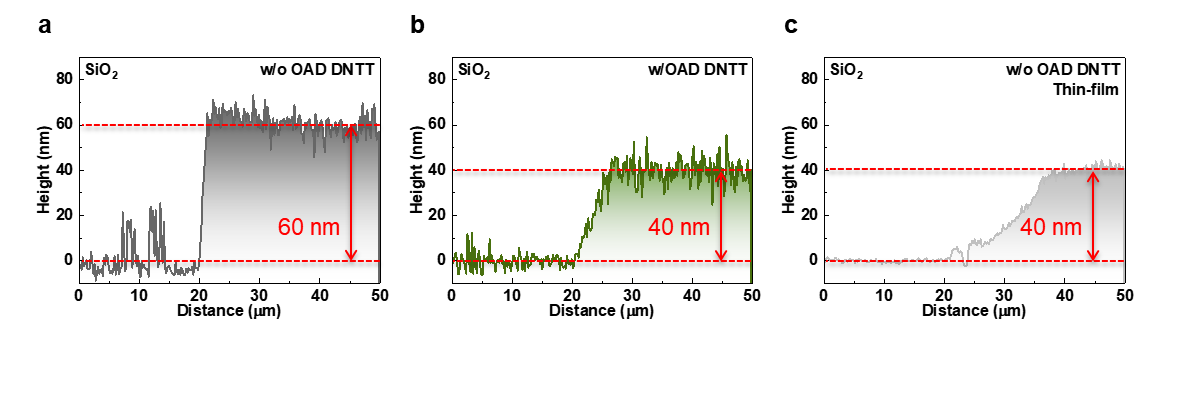
**

**Figure S16.** a) Thickness of the DNTT layer deposited on the non-OAD device. b) Thickness of the DNTT layer deposited on the OAD device. c) Thickness of the non-OAD thin-film device deposited to match the thickness of the OAD device.

**
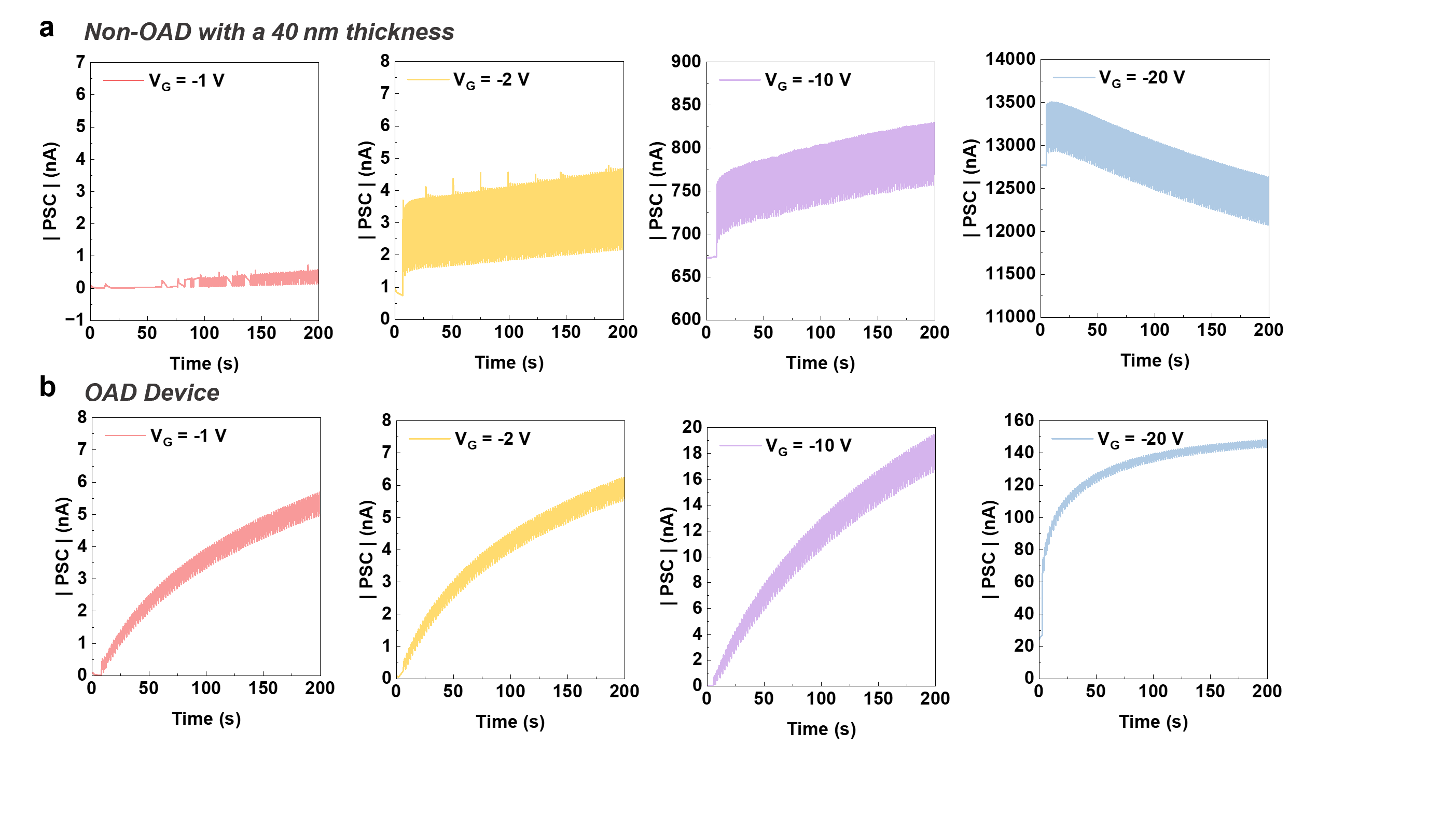
**

**Figure S17.** a) Synaptic characteristics of the non-OAD device with a 40 nm thickness under various *V_G_* values. b) Synaptic characteristics of the OAD device under various *V_G_* values.


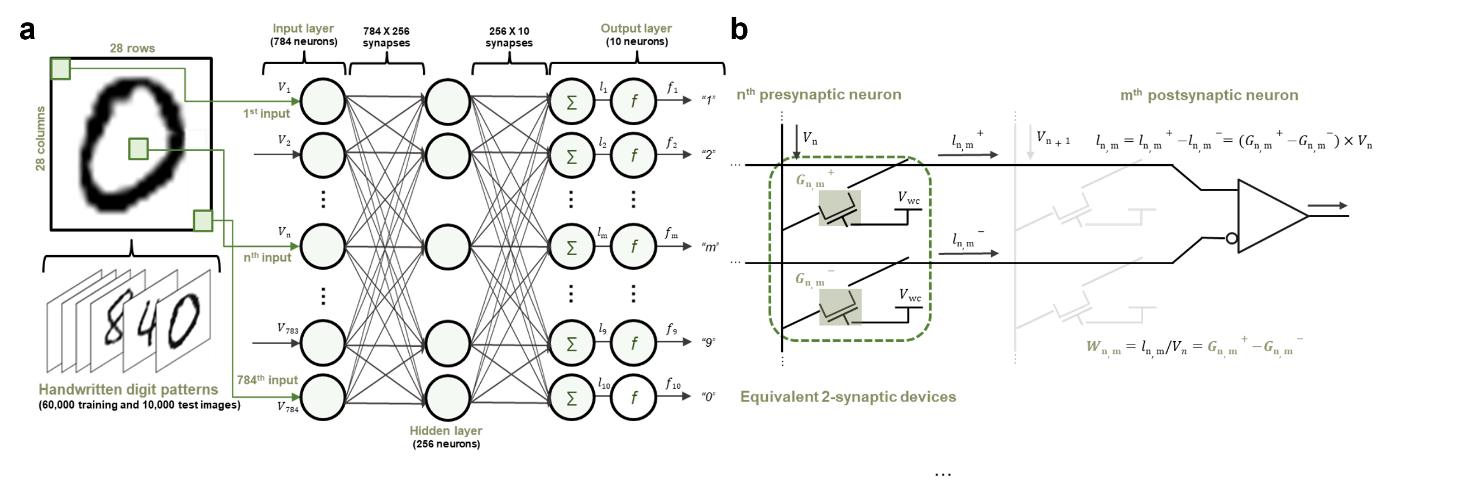


**Figure S18.** a) Schematic diagram of a three-layer ANN for learning MNIST handwritten digit images. b) Synaptic weight is the difference in conductivity between two synaptic devices.


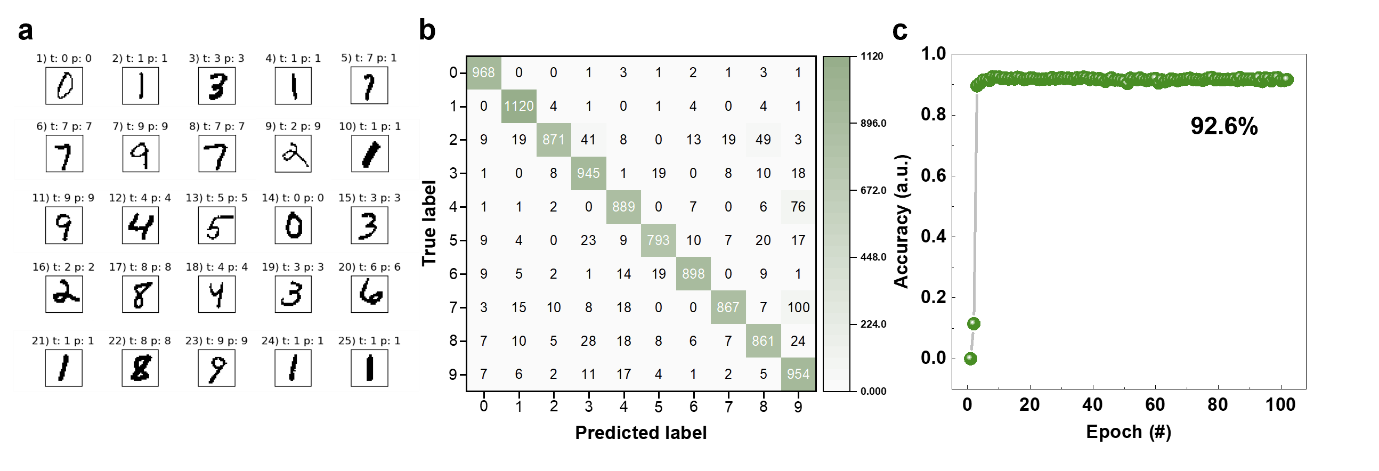


**Figure S19.** MNIST simulation results from the OAD device at *V_G_* = $-$10 V. a) Handwritten digit images with true and predicted labels, classified using the OAD device-based ANN. b) Confusion matrix for ANN, where rows represent the desired output digits, and columns represent the predicted output numbers. Correct classifications are located on the diagonal of the table. c) Training accuracy for handwritten image recognition with respect to different epochs.


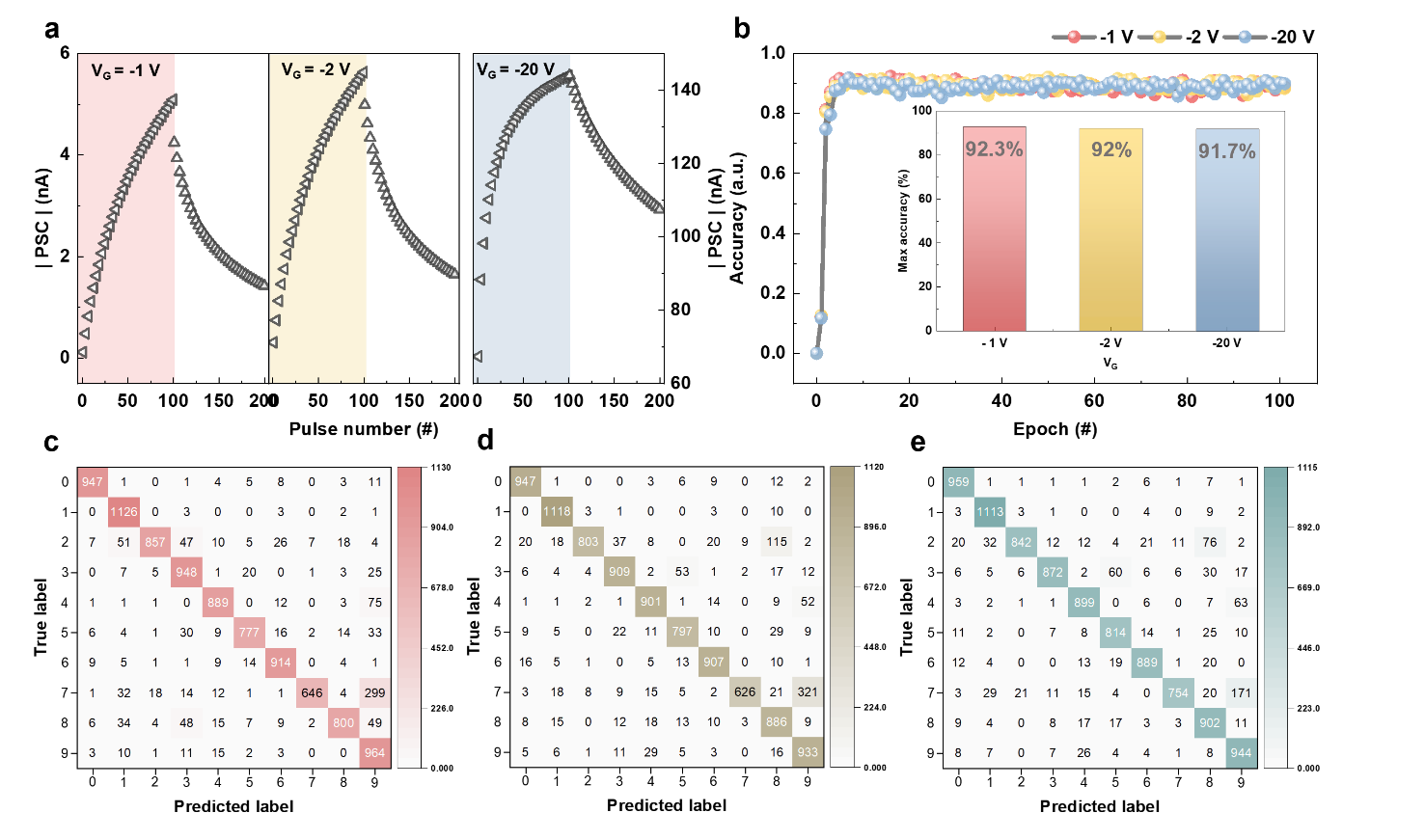


**Figure S20**. a) Potentiation-Depression curves and b) training accuracy for handwritten image recognition with respect to different epochs at *V_G_* = $-$1 V, $-$2 V, and -20 V. c) Confusion matrix at *V_G_* = $-$1 V, d) $-$2 V, and e) $-$20 V.


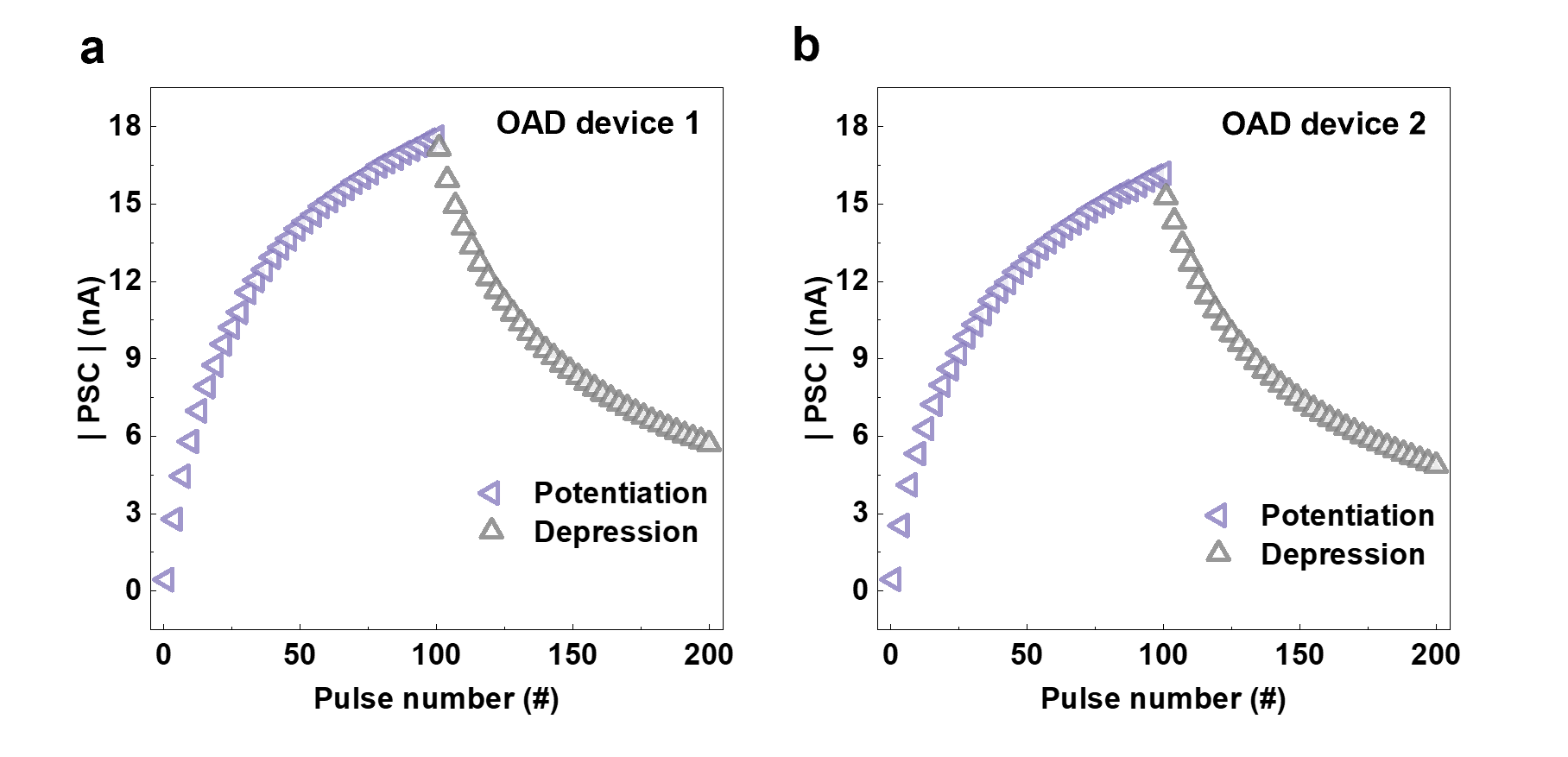


**Figure S21.** PSC values extracted from the potentiation-Depression curve of a) OAD device 1 and b) OAD device 2, respectively.

**Table S1.** Comparison of organic synaptic device characteristics.

| Device structure | Weight-update signals | Dynamic range (Gmax/Gmin) | # of Conductance states (LTP/LTD) | Cycle durability | Recognition accuracy (%) | ANN model/ Train data | [Ref] |
| --- | --- | --- | --- | --- | --- | --- | --- |
| DNTT/ Parylene | Photonic and electrical co-stimulation | >182 | 100/100 | > 52 cycles (5200 pulses) | 92.6 | MLP/ MNIST | This work |
| PDVT-10/PVN | Electrical stimulation | 109.9 | 50/50 | 100 cycles | 85.9 | MLP/ MNIST | [1] |
| IDTBT/PC61BM | Photonic and electrical co-stimulation | 50 | 50/50 | N/A | 86 | SLP/ MNIST | [2] |
| PDPP4T/ NTCDI-F15 | Photonic and electrical co-stimulation | 1080 | 400/400 | N/A | 93 | MLP/ MNIST | [3] |
| PDPP4T/PVP/CsBi3O10 | Photonic and electrical co-stimulation | 1000 | 50/50 | N/A | 91.8 | MLP/ MNIST | [4] |
| C8/CsPbBr3/PS | Photonic and electrical co-stimulation | 40 | 100/100 | 50 cycles | 75 | SLP/ MNIST | [5] |

**Table S2.** Comparison of defect detection accuracy with recent software models.

| Implementation Type | Model | Defect detection accuracy (%) | [Ref] |
| --- | --- | --- | --- |
| HW  (ideal/our device) | MLP | 81.5/75.7 | This work |
| SW | CNN | 92.8 | [6] |
| SW | CNN | 95.5 | [7] |
| SW | YOLOv5 CNN | 95 | [8] |
| SW | CNN | 95.1 | [9] |

**References**

[1] R. Yu, E. Li, X. Wu, Y. Yan, W. He, L. He, J. Chen, H. Chen, T. Guo, *ACS Appl. Mater. Interfaces.* **2020**, 12, 15446.

[2] S. Lan, J. Zhong, J. Chen, W. He, L. He, R. Yu, G. Chen, H. Chen, *J. Mater. Chem. C* **2021**, 9, 3412.

[3] P. Guo, J. Zhang, Z. Hua, T. Sun, L. Li, S. Dai, L. Xiong, J. Huang, *Nano Lett.* **2025**, 25, 3204.

[4] R. Wang, P. Chen, D. Hao, J. Zhang, Q. Shi, D. Liu, L. Li, L. Xiong, J. Zhou, J. Huang, *ACS Appl. Mater. Interfaces.* **2021**, 13, 43144.

[5] Q. Shi, D. Liu, D. Hao, J. Zhang, L. Tian, L. Xiong, J. Huang, *Nano Energy* **2021**, 87, 106197.

[6] V. Mankad, N. Bhanvadia, M. I. Patel, R. Gajjar, presented at *2021 3rd International Conference on Advances in Computing, Communication Control and Networking (ICAC3N)*, **2021**.

[7] L. Lei, H.-X. Li, H.-D. Yang, *IEEE Trans. Instrum. Meas.* **2022**, 72, 1.

[8] A. Bhattacharya, S. G. Cloutier, *Sci. Rep.* **2022**, 12, 12559.

[9] J. Kim, J. Ko, H. Choi, H. Kim, *Sensors* **2021**, 21, 4968.
